# Assessing the accuracy of phylogenetic rooting methods on prokaryotic gene families

**DOI:** 10.1371/journal.pone.0232950

**Published:** 2020-05-15

**Authors:** Taylor Wade, L. Thiberio Rangel, Soumya Kundu, Gregory P. Fournier, Mukul S. Bansal

**Affiliations:** 1 Department of Computer Science & Engineering, University of Connecticut, Storrs, CT, United States of America; 2 Department of Earth, Atmospheric & Planetary Sciences, Massachusetts Institute of Technology, Cambridge, MA, United States of America; 3 Institute for Systems Genomics, University of Connecticut, Storrs, CT, United States of America; Universite de Lausanne Faculte de biologie et medecine, SWITZERLAND

## Abstract

Almost all standard phylogenetic methods for reconstructing gene trees result in *unrooted* trees; yet, many of the most useful applications of gene trees require that the gene trees be correctly rooted. As a result, several computational methods have been developed for inferring the root of unrooted gene trees. However, the accuracy of such methods has never been systematically evaluated on prokaryotic gene families, where horizontal gene transfer is often one of the dominant evolutionary events driving gene family evolution. In this work, we address this gap by conducting a thorough comparative evaluation of five different rooting methods using large collections of both simulated and empirical prokaryotic gene trees. Our simulation study is based on 6000 true and reconstructed gene trees on 100 species and characterizes the rooting accuracy of the four methods under 36 different evolutionary conditions and 3 levels of gene tree reconstruction error. The empirical study is based on a large, carefully designed data set of 3098 gene trees from 504 bacterial species (406 Alphaproteobacteria and 98 Cyanobacteria) and reveals insights that supplement those gleaned from the simulation study. Overall, this work provides several valuable insights into the accuracy of the considered methods that will help inform the choice of rooting methods to use when studying microbial gene family evolution. Among other findings, this study identifies parsimonious Duplication-Transfer-Loss (DTL) rooting and Minimal Ancestor Deviation (MAD) rooting as two of the most accurate gene tree rooting methods for prokaryotes and specifies the evolutionary conditions under which these methods are most accurate, demonstrates that DTL rooting is highly sensitive to high evolutionary rates and gene tree error, and that rooting methods based on branch-lengths are generally robust to gene tree reconstruction error.

## Background

Phylogenetic trees, or phylogenies, represent evolutionary relationships between biological entities. *Gene trees*, which represent the evolutionary relationships between distinct homologs of a gene family, and *species trees*, which represent evolutionary relationships between species (or other groupings of taxa), are the two most widely used kinds of phylogenetic trees. Almost all routinely used methods for constructing such trees result in trees that are *unrooted* [1–4], i.e., there is no information about the direction of evolution or ancestor-descendant relationships; a direct consequence of the commonly used reversible evolutionary models used for phylogeny inference. However, knowledge of how a phylogeny is rooted is fundamental to understanding how genes and species evolve and almost all applications of phylogenies require phylogenetic trees to be correctly rooted. As a result, several techniques have been developed for estimating the correct root position within a tree and these techniques are widely used [5, 6].

Current rooting methods can be broadly classified into four categories. The first category includes methods that are designed specifically for species tree rooting. This category includes outgroup rooting [7–9], which is perhaps the most widely used method for species tree rooting. Outgroup rooting takes advantage of prior knowledge about the taxonomy of the sampled taxa and a known outgroup species (or group of species) is included in analysis; the tree is then rooted at the edge connecting the rest of the tree to this outgroup. Outgroup rooting can also be applied to gene trees of universal (or near-universal) homologs, but it is highly sensitive to evolutionary events such as horizontal gene transfer, duplication, loss, or incomplete lineage sorting. Outgroup rooting therefore cannot be meaningfully used with gene trees that have complex histories of such evolutionary events. Other methods designed for species tree rooting include phylogenomic methods based on phylogenetic reconciliation, where the species tree is rooted based on maximizing evolutionary “fit” with a collection of unrooted gene trees [10–12]. The second category consists of those methods that attempt to locate the root based on branch lengths on the inferred phylogeny. This category includes the very widely used midpoint rooting method [13, 14], where the tree is rooted at the mid point of the longest path in the tree; minimal ancestor deviation (MAD) rooting [15], where the objective is to minimize the mean relative deviation from the molecular clock; and Minimum Variance (MV) rooting [16], where the goal is to minimize root-to-leaf distance variance. The third category consists of those rooting methods that use non-reversible or non-stationary evolutionary models and molecular clock or relaxed molecular clock models to directly infer rooted phylogenies. This category includes strict molecular clock rooting [17], Bayesian molecular clock rooting [18], methods based on non-reversible and/or non-stationary evolutionary models [19–23], and relaxed clock models [24, 25]. Finally, the fourth category of methods are those that are based on phylogenetic reconciliation. These methods are designed specifically for gene tree rooting and work by reconciling an unrooted gene tree with a known rooted species tree. This reconciliation is computed based on the duplication-loss model for eukaryotic gene families [26] and on the duplication-transfer-loss (DTL) reconciliation loss model for prokaryotic gene families [27–31]. Essentially, these methods use the known species tree root to inform the placement of gene tree roots and rely on the fact that species tree root inference is often less complex since it tends to be estimated from more informative and stable data sets.

Even though rooting methods are widely used in practice, the accuracy of most methods has not been systematically evaluated using simulated data sets (but see [16]). In particular, there has never been a systematic evaluation of any of these methods on simulated prokaryotic gene trees (with horizontal gene transfer). Furthermore, to the best of our knowledge, there is only one empirical study that uses prokaryotic data to evaluate the accuracy of some of these rooting methods [15]. As a result, it is not known how well these methods work for rooting prokaryotic gene trees, where horizontal gene transfer is ubiquitous.

In this work we address this gap by conducting an extensive comparative evaluation using both simulated and empirical bacterial gene trees. We focus on evaluating methods from the second and fourth categories of rooting methods above; specifically midpoint rooting, MAD rooting, and MV rooting from the second category, and parsimonious DTL rooting and ALE rooting (i.e., likelihood-based DTL rooting) from the fourth category. We exclude from consideration methods tailored for species tree rooting (first category) and not directly applicable to prokaryotic gene trees. We also exclude from consideration all methods from the third category since they are highly computationally intensive and are seldom used in practice; furthermore, methods from the third category rely on tree reconstruction from sequence data, resulting in topological inconsistencies that make it difficult to compare rooting accuracy across different methods.

Our simulation study, based on 3600 true (i.e., error-free) gene trees and 2400 reconstructed (i.e., error-prone) gene trees on 100 species, characterizes the rooting accuracy of the five chosen methods under a wide range of evolutionary conditions. While these simulations do not adequately capture the complexities of real data, they serve as a proof of concept of how each rooting method performs under a controlled setting and inform our expectations about how the methods might behave on empirical data sets. We find that, (parsimonious) DTL rooting performs exceptionally well when the gene tree error rate is low and the rate of evolutionary events such as gene duplication, loss, and horizontal gene transfer is low to moderate, but that its performance degrades rapidly for increasing rates of evolutionary events and gene tree error. In fact, we find that as the number of transfer events increases to very high levels, DTL rooting generally prefers highly incorrect and unbalanced rootings. In contrast, rooting accuracy of the other three methods considered was only mildly affected by increasing rates of these evolutionary events and, surprisingly, were remarkably robust to gene tree reconstruction error. On the other hand, while DTL rooting is unaffected by deviations from the molecular clock, the rooting accuracy of all the other methods is affected significantly as deviation increases. Surprisingly, we find that ALE rooting performs consistently worse than DTL rooting even though both methods are based on duplication-transfer-loss reconciliation and ALE performs inference under a more complex probabilistic model of gene evolution. We also find that the specific parameters (event costs) used for DTL rooting can have a significant impact on rooting accuracy, with rooting accuracy increasing when higher costs (than generally used by default) are used for transfer events. Among MAD rooting, MV rooting, and midpoint rooting, no method clearly outperformed the others in the simulation study.

Our empirical analysis uses a large, carefully designed data set of 3098 gene trees from 504 bacterial species divided between 406 Alphaproteobacteria and 98 Cyanobacteria. Note that it is not possible to directly evaluate the accuracy of each rooting method on empirical data given the innate uncertainty of evolutionary reconstruction. This data set was therefore designed such that the placement of the root in gene families conserved in both phyla likely coincides with taxonomic boundaries, i.e., is likely to be placed between Alphaproteobacteria and Cyanobacteria. In particular, while the gene trees in this data set frequently show extensive incongruence with the species tree, corresponding to high frequencies of DTL events, horizontal gene transfers between Alphaproteobacteria and Cyanobacteria accounted for a very small fraction of gene transfers among gene families present in both phyla. Thus, on this data set, we assessed rooting similarities among methods by comparing the placement of each proposed gene tree root with the expected placement given the overall balance of the taxonomic distribution between Cyanobacteria and Alphaproteobacteria (0.2413). We found that root positions inferred by MAD and MV rooting showed the most agreement among all assessed methods, while DTL rooting and ALE rooting yielded the most divergent root positions compared to MAD and MV rooting. Balance-wise, root positions estimated by MAD rooting were closest to the expected placement, with MV rooting showing similar performance, while root positions obtained by DTL rooting and ALE rooting were frequently far from the expected position and phylogenetically shallow.

Overall, our simulation and empirical studies suggest that: (1) Among the methods tested, DTL rooting and MAD rooting may be the two most effective methods for rooting prokaryotic gene families. Specifically, we find that DTL rooting outperforms branch-length based methods for low to moderate rates of evolutionary events, even at relatively low (but not zero) deviation from the molecular clock, while MAD rooting is most accurate for higher rates of evolutionary events, even with high levels of molecular clock deviation. (2) ALE rooting consistently performs worse than DTL rooting. (3) MV rooting and MAD rooting have similar rooting behaviors. (4) DTL rooting becomes substantially more accurate when a higher cost is used for transfer events than is generally used by default. (5) DTL rooting can be positively misleading, approaching the accuracy of random rooting, when applied to gene trees with very high rates of horizontal gene transfer. And (6), MAD, MV, and midpoint rooting are generally robust to gene tree reconstruction error and remain effective at rooting even when the gene trees under consideration have significant reconstruction error, while DTL rooting is more sensitive to such errors.

## Materials and methods

### Simulated data sets

To systematically test the rooting accuracy of the five tested methods we generated 3600 simulated gene trees using the SaGePhy [32] software package. These simulated gene trees represent a wide range of evolutionary conditions and we grouped them into three broad collections of data sets. The first collection, referred to as the *baseline data sets*, consists of 12 sets of gene trees, with each set containing 100 gene trees. The 12 sets represent three different rates (Low, Medium, and High) of duplication, transfer, and loss events, combined with four different schemes for scaling gene tree branches. The second collection, which we call the *additive data sets*, is designed to test if the type of transfer event, additive transfer or replacing transfer, used in the simulation affects rooting accuracy of the tested methods. Both additive and replacing transfers occur in genome evolution, with the former corresponding to novel gene acquisitions, and the latter being balanced by a loss of an orthologous native copy of the gene. This is also often called “xenologous gene displacement” [33]. The additive data sets consist of 12 sets of gene trees just as in the baseline collection, with the only difference being that all transfer events used to generate the additive data sets are additive transfers, while a 3:7 ratio was used for additive and replacing transfers in the baseline data sets. The third collection, which we refer to as the *very high transfer rate data sets*, is designed to test how very high prevalence of transfer events affects rooting accuracy of the tested methods. These very high transfer rate data sets consist of 12 sets of gene trees, with each set containing 100 gene trees, representing three different rates of transfer events combined with four different branch-scaling schemes.

In addition to the 3600 simulated gene trees above, we used the 1200 gene trees from the baseline data sets to evolve sequences and create reconstructed gene trees with errors. This fourth collection, called *reconstructed baseline data sets*, is designed to test the impact of gene tree reconstruction error on the accuracy of root inference. These reconstructed baseline data sets consist of 24 sets of gene trees, with each set having 100 reconstructed gene trees, corresponding to two different reconstruction error rates, referred to as *low error* and *high error*, for each of the 12 baseline sets of gene trees.

Thus, the simulated data sets consist of a total of 6000 true and reconstructed gene trees. We provide further details on the construction of these 6000 gene trees below.

#### Baseline data sets

We first evolved 100 species trees, each with exactly 100 taxa and a height of 1, using a probabilistic birth-death process. We then evolved 100 low-DTL gene trees, 100 medium-DTL gene trees, and 100 high-DTL gene trees by evolving three gene trees inside each of these 100 species trees using low, medium, and high rates of duplication, transfer, and loss events. The three resulting sets of gene trees were each scaled using four different branch-scaling schemes, yielding a total of 12 sets of 100 gene trees each. The specific parameters used are as follows:

- Gene tree simulation:
  - For low rates of DTL, the duplication rate was equal to 0.13 and total transfer rate equal to 0.27. For medium rates of DTL, the duplication rate was equal to 0.3 and total transfer rate equal to 0.6. For high rates of DTL, the duplication rate equaled 0.6 and total transfer rate equal to 1.2. These rates are specified in terms of expected number of events per unit branch length and are based on rates observed in real data; they capture data sets with lower rates of these events as well as data sets with high rates of these events [34]. In each case the loss rate was set to be equal to 0.8 × (duplication rate + additive transfer rate). This loss rate appropriately models slowly expanding gene families, and leads to the creation of both small and large gene families.
  - The ratio of additive to replacing transfers was fixed at 3:7, reflecting the observation that in many biological scenarios replacing transfers are more frequent then additive transfers [35].
- Branch length scaling:
  - Uniform random scaling in the range [0.3, 3], with each branch scaled independently.
  - Uniform random scaling in the range [0.2, 5], with each branch scaled independently.
  - Autocorrelated lognormal rate scaling using the model of Rannala and Yang [36], with start rate 1 and *σ*^2^ = 0.05.
  - Autocorrelated lognormal rate scaling using the model of Rannala and Yang [36], with start rate 1 and *σ*^2^ = 0.25.

#### Additive data sets

These data sets were generated identically to the baseline data sets above, except that all transfer events in the simulation were fixed to be additive transfer events (i.e., the gene trees did not contain any replacing transfers).

#### Very high transfer rate data sets

To further assess the impact of transfers on rooting accuracy, we simulated additional data sets with very high rates of transfer events. These data sets were simulated similarly to the baseline data sets, except that instead of low-DTL, medium-DTL, and high-DTL rates, we have *very high 2*, *very high 3*, and *very high 4* data sets. The specific event rates used are given below.

- For the very high 2, very high 3, and very high 4 data sets, the total transfer rate was fixed at 2.0, 3.0, and 4.0, respectively. In each case, the duplication rate corresponded to the high-DTL rate of baseline data sets, i.e, the duplication rate equaled 0.6. Likewise, in each case the loss rate was set to be equal to (0.8 × (duplication rate + additive transfer rate)).
- As in the baseline data sets, the ratio of additive to replacing transfers was fixed at 3:7.

Branch scaling was done identically as for the baseline data sets, yielding a total of 12 sets with 100 gene trees each.

Table 1 shows the average leaf set size, and average numbers of duplications, losses, additive transfers, and replacing transfers for the simulated gene tree sets from each of the three collections of data sets.

**Table 1:** Simulated data sets. This tables shows the average numbers of leaves, duplication events, losses, additive transfer events, and replacing transfer events in the various baseline, additive, and very high transfer rate data sets. Results are averaged across the 100 gene trees in each set.

	Leaves	Duplications	Losses	Additive Transfers	Replacing Transfers
Baseline Low-DTL	118.49	3.23	1.34	1.77	3.81
Baseline Medium-DTL	108.89	5.73	8.35	3.38	7.98
Baseline High-DTL	121.05	11.72	18.13	6.79	14.6
Additive Low-DTL	105.77	2.4	7.02	5.58	0
Additive Medium-DTL	111.25	5.16	15.56	11.84	0
Additive High-DTL	140.61	12.33	38.34	24.95	0
Very high 2	128.26	11.11	24.2	9.87	23.46
Very high 3	136.35	10.85	31.43	14.7	32.54
Very high 4	130.7	10.11	36.07	17.45	36.81

#### Reconstructed baseline data sets

To simulate gene trees with phylogenetic reconstruction errors, we used the 1200 true gene trees from the baseline data sets to evolve nucleotide sequences of length 1000nt and 500nt using Seq-Gen [37]. We then used RAxML [2] to reconstruct gene trees using the resulting nucleotide sequences. Thus, for each gene tree from the baseline data set, we created two corresponding reconstructed gene trees; one with a low error rate, corresponding to the longer 1000nt sequences, and another with a high error rate, corresponding to the shorter 500nt sequences. This resulted in a total of 2400 reconstructed gene trees corresponding to the baseline data sets (1200 low-error reconstructed trees and 1200 high-error reconstructed trees).

Table 2 shows the average Robinson-Foulds distance (defined formally in the Section on “Measurement of rooting accuracy”) for the 24 sets of reconstructed baseline gene trees. Specific Seq-Gen and RAxML commands used appear in S1 Appendix.

**Table 2:** Error-rates for reconstructed gene trees. Average Robinson-Foulds distance for the 12 sets of low-error reconstructed baseline gene trees and the 12 sets of high-error reconstructed baseline gene trees. Results are averaged across the 100 gene trees in each set. The table also shows the average number of leaves in each data set.

Baseline set	Leaves	Low-Error Norm. RF Distance	High-Error Norm. RF Distance
Low-DTL, Uniform [0.3, 3]	118.49	7.77	12.24
Medium-DTL, Uniform [0.3, 3]	108.89	7.64	11.46
High-DTL, Uniform [0.3, 3]	121.05	7.95	12.23
Low-DTL, Uniform [0.2, 5]	118.49	10.79	15.46
Medium-DTL, Uniform [0.2, 5]	108.89	9.9	13.67
High-DTL, Uniform [0.2, 5]	121.05	10.37	15.25
Low-DTL, Auto. [1, 0.05]	118.49	5.68	8.79
Medium-DTL, Auto. [1, 0.05]	108.89	5.39	8.47
High-DTL, Auto. [1, 0.05]	121.05	5.91	9.09
Low-DTL, Auto. [1, 0.25]	118.49	5.43	8.76
Medium-DTL, Auto. [1, 0.25]	108.89	5.41	8.28
High-DTL, Auto. [1, 0.25]	121.05	5.76	9.47

### Biological data set

The empirical data set is composed of annotated protein sequences from 504 complete genomes from two distinct Bacteria phyla, Alphaproteobacteria and Cyanobacteria. Limiting the data set to two distinct phyla aims to minimize the impact of inter-phylum HGT events, and as such provide good metrics for rooting accuracy. All annotated gene products were obtained from NCBI’s RefSeq database [38]. Homolog clustering was performed by generating networks of protein sequences connected by E-value weighted edges and applying a Markov Clustering algorithm [39]. E-values were obtained from all-vs-all pairwise USearch comparisons with a threshold of 10^−5^ and using a “negative log-10 transformation” as suggested in [39]. All 3098 homolog groups present in more than 100 taxa were aligned using MAFFT [40] (“–auto –reorder”) with phylogenies reconstructed using IQTree [41] (“-m MFP -mset LG,WAG,BLOSUM62 -mrate G -mfreq F”).

All simulated and biological data sets used in this study are available freely from https://compbio.engr.uconn.edu/datasets.

### Description of rooting methods evaluated

We tested five methods appropriate for rooting prokaryotic gene trees. These are briefly described below.

#### Midpoint rooting

In this method, the unrooted tree under consideration is rooted at the mid point of the longest path in the tree [13, 14]. This is one of the oldest and most widely used rooting method for gene trees (see, e.g., [6]).

#### Minimal Ancestor Deviation (MAD) rooting

The MAD rooting method works by considering all edges on the unrooted tree as a possible root position and calculating the mean relative deviation from the molecular clock implied by each such rooting [15]. It then roots the tree at the edge that minimizes this relative deviation.

#### Minimum Variance (MV) rooting

MV rooting is a recently developed method [16] intended to improve upon midpoint rooting. While midpoint rooting essentially seeks a root that minimizes the largest root-to-leaf distance, the MV rooting method finds a root that minimizes root-to-leaf distance variance.

#### Parsimonious Duplication-Transfer-Loss reconciliation (DTL) rooting

DTL rooting [30, 31] is designed specifically for rooting prokaryotic gene trees and is based on parsimoniously reconciling an unrooted gene tree with a known rooted species tree. It accounts for some of the major evolutionary processes responsible for gene tree/species tree discord; specifically gene duplications, gene losses, and horizontal gene transfers. This method computes the minimum reconciliation cost against the species tree for all possible rootings of the gene tree and assigns a root that minimizes this reconciliation cost. DTL rooting uses a parsimony framework and requires as input specific event costs for duplications, transfers, and losses. In this work, we consider four different event cost assignments to study their impact on rooting accuracy. By default, we use the default costs implemented in the OptRoot program from the RANGER-DTL software package [31], which are 1, 2, and 3, respectively, for losses, duplications, and transfers. These costs have been shown to work well for inferring reconciliations under a wide range of evolutionary scenarios [34]. In addition, we also used 〈*loss, duplication, transfer*〉 costs of 〈1, 1, 2〉, 〈1, 2, 4〉, and 〈1, 2, 5〉. Since accurate species tree dating remains a difficult problem in practice, in our study, DTL rooting was applied using undated species tree.

#### Amalgamated Likelihood Estimation (ALE) rooting

Like DTL rooting above, ALE rooting [27] is designed specifically for prokaryotic gene trees and is based on a probabilistic model of gene family evolution inside a given rooted species tree. The primary purpose of this method is gene tree error-correction, but it can also be used just for rooting a given unrooted gene tree. ALE takes into account gene duplications, gene losses, and horizontal gene transfers and seeks error-corrected and rooted reconciled gene trees that maximize likelihood under the probabilistic evolutionary model. In this work, we use ALE only for gene tree rooting, not for gene tree error-correction. We used the undated version of ALE (*ALEml_undated*), which does not require a dated species tree, when evaluating ALE rooting on the simulated and real data sets.

It is worth noting that midpoint rooting, MAD rooting, and MV rooting critically use gene tree branch lengths to identify roots, while DTL rooting and ALE rooting ignore branch lengths and only use gene tree topologies (along with corresponding species tree topologies) for root identification. Not using branch lengths for gene tree rooting has both advantages and disadvantages. Gene tree branch lengths are directly impacted by substitution rate variation along tree edges, which can mislead methods that depend on branch-lengths for rooting. Branch lengths can also be difficult to infer accurately. On the other hand, branch lengths represent a valuable source of information about evolutionary distances and methods that ignore branch lengths are unable to make use of this information.

#### Comparison against random rootings

To fully assess the performance of the methods above, we also rooted the gene trees at random (at a randomly chosen edge of the unrooted tree).

### Measurement of rooting accuracy

To assess rooting accuracy we used two easily interpretable measures. For each combination of rooting method and gene tree set (36 sets of 100 gene trees each), we measured (i) the total number of gene trees (out of 100) that were rooted correctly, and (ii) the average Robinson-Foulds (RF) distance [42] between each correctly rooted gene tree and the corresponding gene tree with inferred rooting. To compute the RF distance between two rooted trees *T*_1_ and *T*_2_ on the same set of leaves, denoted *RF*(*T*_1_, *T*_2_), we first compute the number of *clades* (or *clusters*) found in only one of the trees and not in the other, and then divide this count by 2 (since the distance is symmetric in the two trees being compared). The choice of using RF distance for measuring rooting accuracy was informed by the following observation.

**Observation 1** *Let T*_1_ *and T*_2_ *be rooted versions of the same unrooted tree T, and let (u*_1_, *u*_2_) *and (v*_1_, *v*_2_) *denote the edges of T on which T*_1_ *and T*_2_ *are rooted, respectively. Without loss of generality, assume that the path in T from u*_1_ *to v*_1_ *is longer than the paths from u*_1_ *to v*_2_, *u*_2_ *to v*_1_, *and u*_2_ *to v*_2_. *Then, RF(T*_1_, *T*_2_) *must be equal to the number of intermediate nodes on the path between u*_1_ *and v*_1_ *in T*.

**Fig 1:**
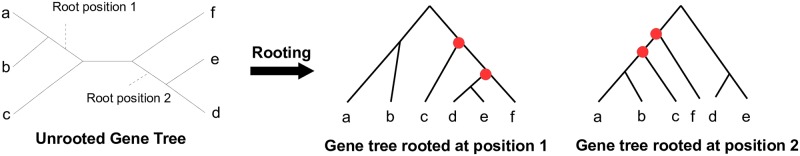
Relationship between alternative rootings and RF distance. The two rooted gene trees, shown on the right, are obtained by rooting the unrooted gene tree on the left along its two marked edges. The shaded (red) nodes on these two rooted trees mark those clades that appear only in one of the rooted trees and not in the other. Thus, the RF distance between these two rooted trees is 2, corresponding to the distance (number of nodes) between the two marked root positions on the unrooted tree.

The observation above, illustrated in Fig 1 through an example, implies that RF distance exactly captures the “distance” between two alternative rootings of the same unrooted tree.

In addition to computing these “absolute” RF distances, as described above, we also compute and report *normalized* RF distances where the absolute RF distance is divided by the number of leaves in the corresponding gene tree. This normalized RF distance captures deviation from the true root position relative to gene tree size.

#### Rooting accuracy for reconstructed gene trees

Assessing rooting accuracy for reconstructed gene trees is a little more challenging since it can be difficult to define the “correct” root on an incorrect gene tree. However, we can leverage the observation above to define a meaningful measure of rooting accuracy based on comparing the correctly rooted true tree and a rooted reconstructed tree. In particular, if *T*_1_ denotes the correctly rooted true gene tree, *T*_2_ denotes a rooted reconstructed gene tree (corresponding to *T*_1_), and *URF*(*T*_1_, *T*_2_) denotes the *unrooted* RF distance between unrooted versions of *T*_1_ and *T*_2_, then we must have *RF*(*T*_1_, *T*_2_)≥*URF*(*T*_1_, *T*_2_). If *T*_2_ is “correctly” rooted then *RF*(*T*_1_, *T*_2_) = *URF*(*T*_1_, *T*_2_), while the value of *RF*(*T*_1_, *T*_2_) would keep increasing as *T*_2_ is rooted more and more incorrectly. More precisely, *RF*(*T*_1_, *T*_2_) − *URF*(*T*_1_, *T*_2_) captures the distance between the roots of *T*_1_ and *T*_2_, even when *T*_1_ and *T*_2_ are not identical. We refer to *RF*(*T*_1_, *T*_2_)−*URF*(*T*_1_, *T*_2_) as the *adjusted RF distance* and use it to measure the relative rooting accuracies of the different methods on the same reconstructed gene trees.

#### Rooting accuracy for empirical gene trees

For the empirical data set, although we do not know the true root position of each gene family *a priori*, the average “root balance ratio” provides a proxy metric for rooting accuracy as the evolutionary signal of inter-phyla gene exchange is not expected to overcome that of intra-phylum exchange”. This root balance ratio is defined to be the ratio of the sizes of the two clades (specifically, the number of leaves in the smaller clade divided by number of leaves in the larger clade) connected at the inferred root, and we expect correctly rooted gene trees to have a similar root balance ratio as the species tree.

#### Handling multiple optimal root positions

Among the tested methods, DTL rooting, MAD rooting, and ALE rooting can sometimes result in multiple (i.e., non-unique) optimal root positions. For our simulated data analysis, we selected an optimal gene tree root arbitrarily whenever there were multiple root candidates. For the empirical study, we chose the optimal rooting with most favorable root balance for MAD and DTL rooting, and chose the most frequent rooting among 100 samples for ALE rooting.

## Results

We applied all five rooting methods to the baseline simulated data sets and real data sets and found that ALE rooting performed consistently worse than DTL rooting on both simulated and real data. Specifically, for the 12 baseline simulated data sets, we found that ALE rooting had worse average absolute RF distances across all 12 data sets, with an overall average of 0.825 compared to 0.625 for DTL rooting. ALE rooting also required longer running times than DTL rooting. In the remainder of this section, we therefore report detailed results only for MAD rooting, MV rooting, midpoint rooting, and DTL rooting. For completeness, results for ALE rooting appear in Tables A1 and A2 and Figs A6, A8, and A9 in S1 Appendix.

### Results of simulation study

#### Baseline data sets

**Fig 2:**
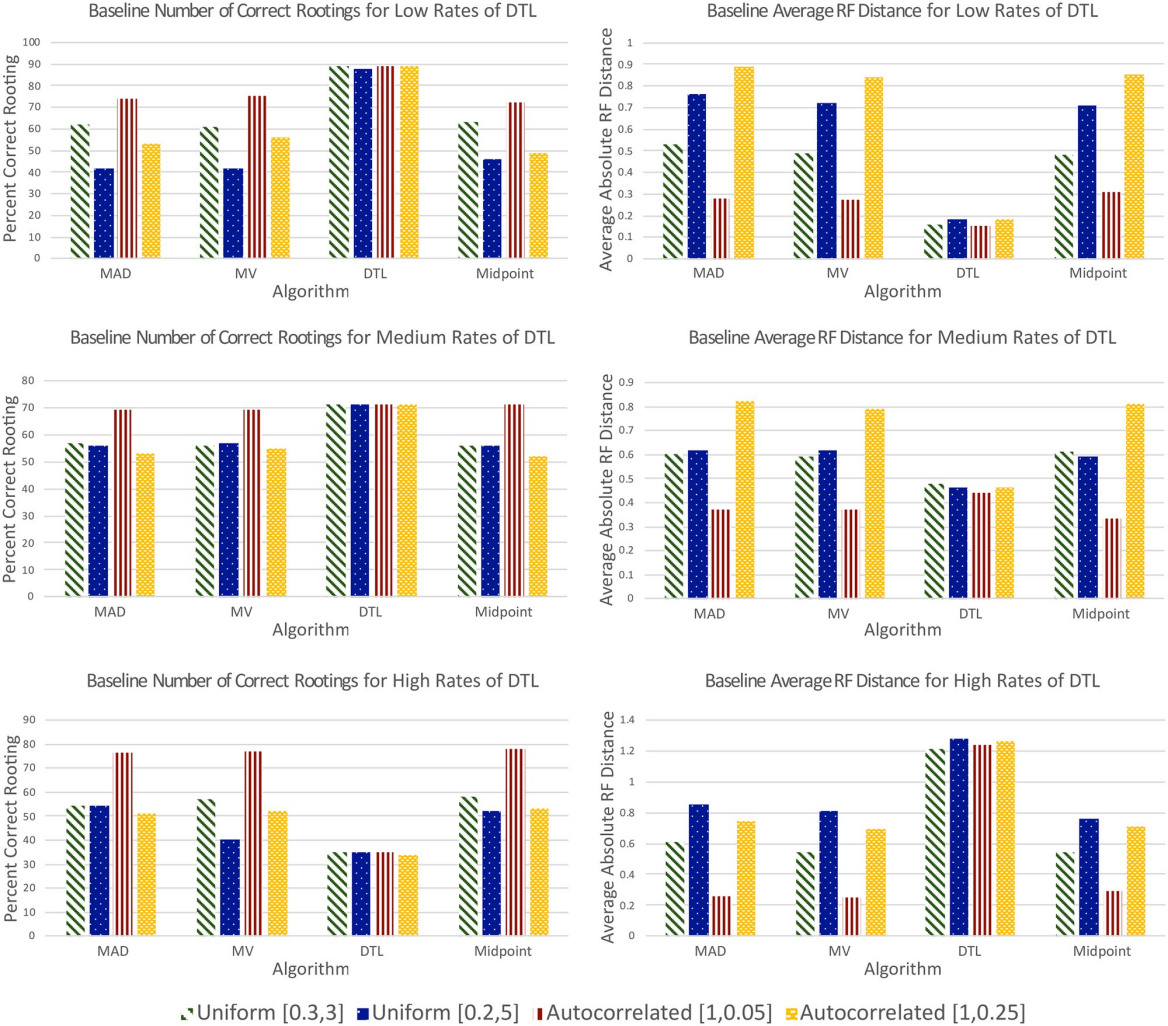
Results on baseline data sets. The plots on the left show, for each rooting method and each of the 12 baseline data sets, the percent of gene trees whose roots were inferred correctly. The corresponding plots on the right show the absolute average RF distances for the rooting methods and data sets. As shown, DTL rooting is most accurate on the low and medium DTL data sets and is unaffected by substitution rate variation along edges (i.e., by branch length scaling). MAD, MV, and midpoint rooting are largely unaffected by DTL event rates but are significantly affected by substitution rate variation along edges; these three rooting methods show comparable accuracy on the baseline data sets. ALE rooting performed worse than DTL rooting across all data sets (results shown in Tables A1 and A2 in S1 Appendix).

Fig 2 shows the accuracy of each method on the 12 baseline data sets. These results show that DTL rooting (using default event costs) significantly outperforms all other methods for all low-DTL data sets, and also clearly outperforms all other methods for the majority of medium-DTL data sets. Specifically, the average absolute RF distances for DTL rooting, MAD rooting, MV rooting, and midpoint rooting across all four branch scalings are 0.17, 0.61, 0.58, and 0.59 for the low-DTL data sets, and 0.46, 0.60, 0.59, and 0.58 for the medium-DTL data sets, respectively. Likewise, the percentage of gene trees that were rooted correctly by DTL rooting, MAD rooting, MV rooting, and midpoint rooting across the four branch scalings are 88.75, 57.75, 58.5, and 57.5 for the low-DTL data sets, and 71, 58.75, 59.25, and 58.75 for the medium-DTL data sets, respectively. However, the accuracy of DTL rooting decreases rapidly as the rate of evolutionary events increases and DTL rooting becomes the worst performing method for high-DTL data sets; the average absolute RF distances for DTL rooting, MAD rooting, MV rooting, and midpoint rooting for the high-DTL data sets across all four branch scalings are 1.25, 0.61, 0.57, and 0.57, respectively. Note that the accuracy of DTL rooting is not affected by branch scaling. We also point out that these results for DTL rooting rely on the availability of a true or materially accurate species tree. While accurate species trees can be constructed in many cases, the accuracy of DTL rooting is expected to degrade if an incorrect species tree is used.

In contrast to DTL rooting, results show that the accuracies of MAD, MV, and midpoint rooting are generally unaffected by the rate of DTL events and remain roughly the same across the low, medium, and high-DTL data sets. Furthermore, while the accuracy of DTL rooting is unaffected by branch length scaling, the accuracies of MAD, MV, and midpoint rooting are significantly affected by substitution rate variation along edges. Averaging across all three event rates, the average absolute RF distances for MAD rooting, MV rooting, and midpoint rooting are 0.58, 0.54, and 0.62 for the data sets with [0.3, 3] uniform random scaling, 0.74, 0.72, and 0.69 for the data sets with [0.2, 5] uniform random scaling, 0.30, 0.30, and 0.31 for the data sets with [1, 0.05] autocorrelated scaling, and 0.82, 0.77, and 0.79 for the data sets with [1, 0.25] autocorrelated scaling. Thus, MAD, MV, and midpoint rooting have similar overall accuracies on these data sets (though the accuracy of MV rooting seems slightly better than that of MAD and midpoint rooting overall). Normalized RF distances for all methods are reported in Table A1 in S1 Appendix, and the three quartiles of absolute RF distances for each method on each simulated baseline data set are reported in Table A2 in S1 Appendix.

These results also show that, in the majority of cases (and except for DTL rooting), [1, 0.25] autocorrelated branch-length scaling yields the most inaccurate rootings while [1, 0.05] autocorrelated scaling yields the most accurate rootings. This is not surprising since scaling under the latter causes only a slight deviation from a global molecular clock, while scaling under the former causes a larger deviation.

**Fig 3:**
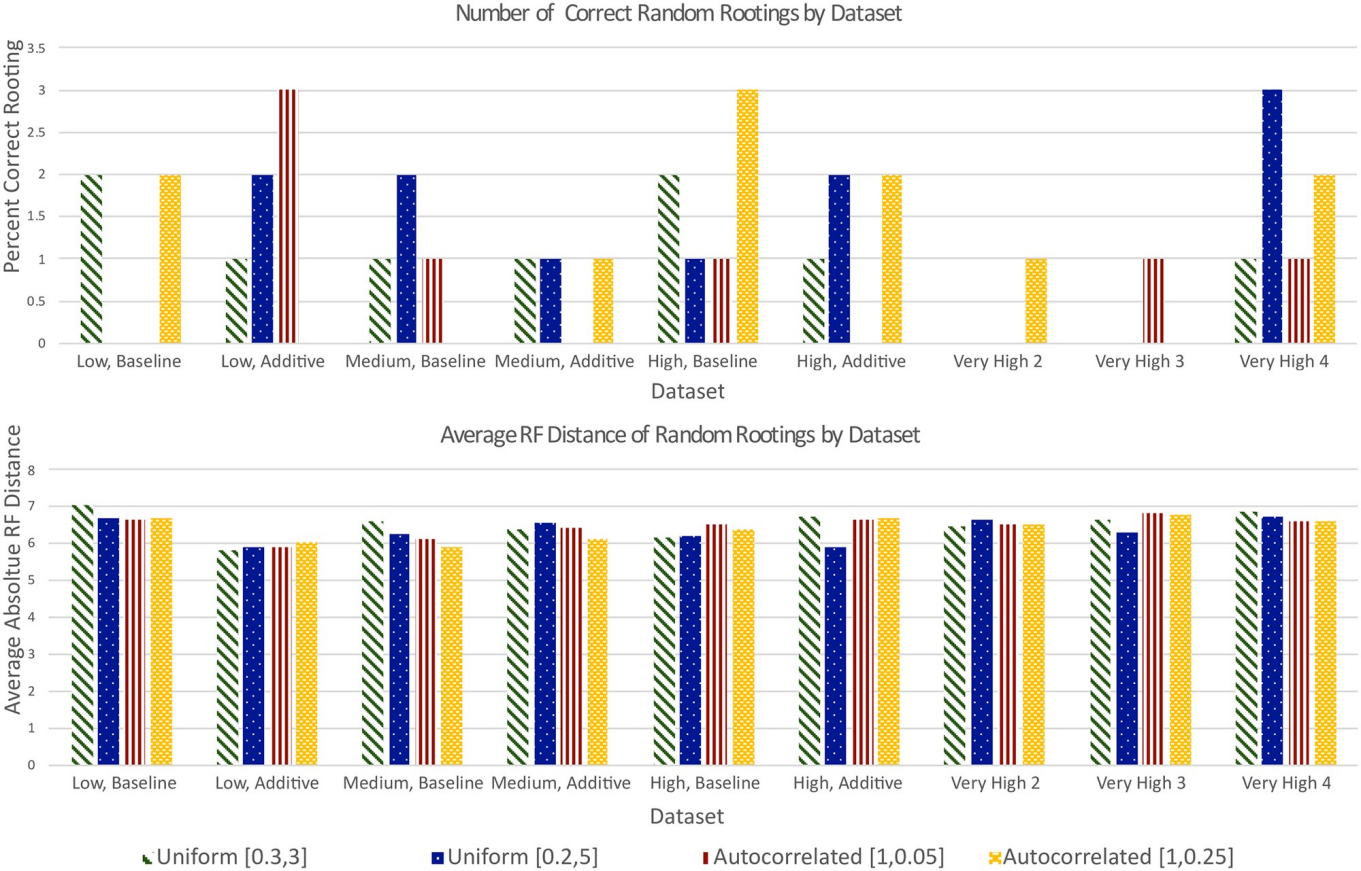
Performance of random rooting on true simulated gene trees. The top half shows, for each set of 100 gene trees, the percent of gene trees for which the root was inferred correctly. The bottom half shows, for each set of gene trees, the absolute average RF distance of the inferred rooted gene trees to the correctly rooted gene trees. As expected, random rooting is unaffected by increasing rates of evolutionary events, by relative abundance of additive and replacing transfers, or by substitution rate variation (or scaling) across branches. The average absolute RF distance for random rooting roughly varied between 6 and 7 for each of the 36 evolutionary conditions.

In contrast with random rooting (Fig 3), all five rooting methods produce far more accurate rootings on all baseline data sets.

#### Additive data sets

As Figs A1 through A4 in S1 Appendix show, the overall accuracy of the four rooting methods is unaffected by the relative abundance of additive to replacing transfers and remains similar to that of the baseline data sets. Thus, the methods are equally robust to additive and replacing transfers.

#### Very high transfer rate data sets

**Fig 4:**
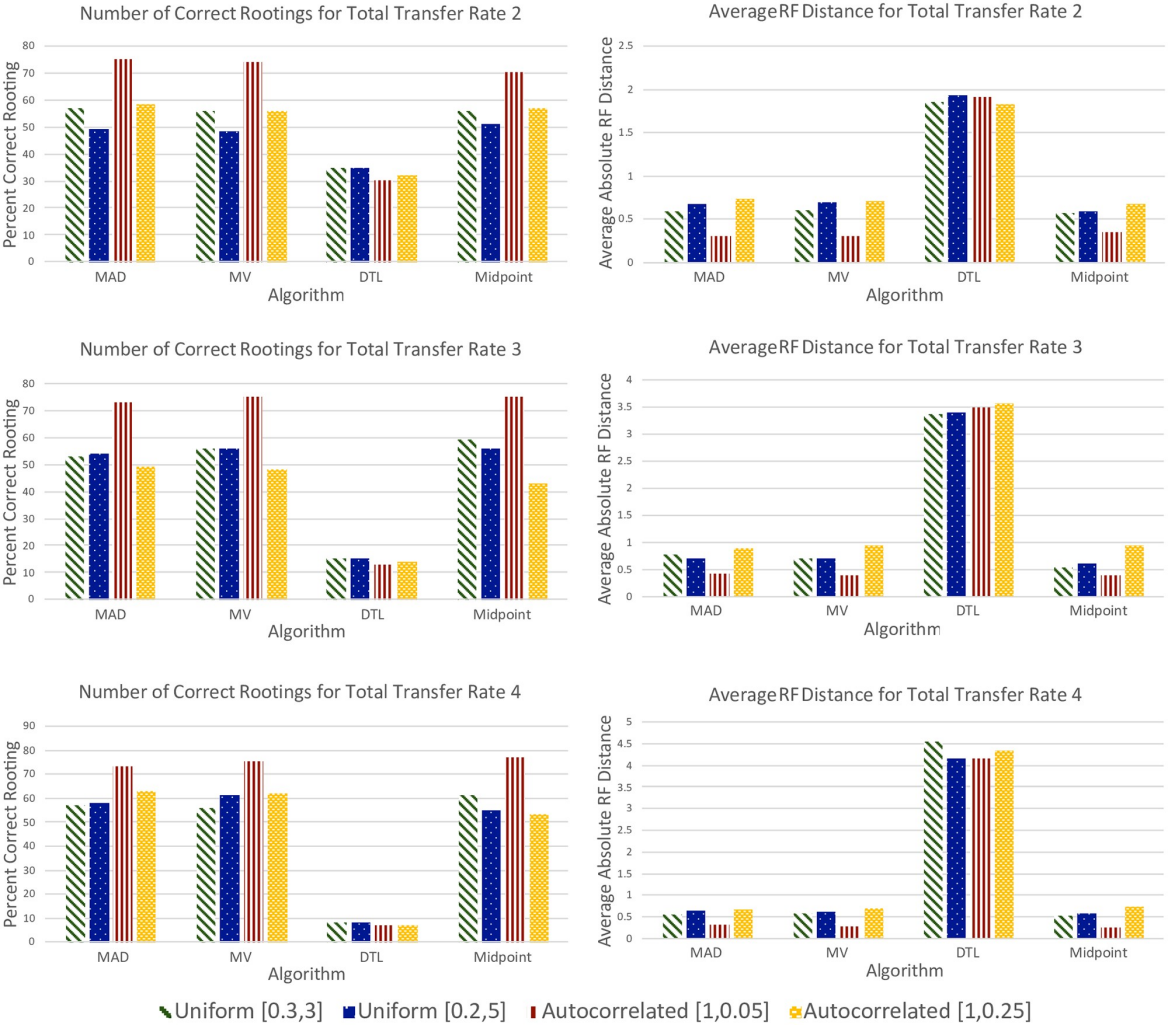
Results on very high transfer rate data sets. As the plots show, MAD, MV, and midpoint rooting are robust to increasing transfer rates and show comparable accuracy on the 12 very high transfer rate data sets. On the other hand, DTL rooting, while unaffected by substitution rate variation along edges, becomes progressively worse as the transfer rate increases and infers far worse root positions than the other three rooting methods.

Our results on these data sets (Fig 4) show that MAD, MV, and midpoint rooting continue to remain robust to increasing transfer rates, but that the performance of DTL rooting suffers dramatically as the transfer rate increases. In fact, as Fig 3 shows, DTL rooting performance starts to approach that of random rooting on these data sets, inferring highly skewed roots that yield highly unbalanced trees. This tendency of DTL rooting to infer skewed/unbalanced rootings is surprising and suggests that DTL rooting should not be used when the rate of evolutionary events is very high.

#### Reconstructed baseline data sets

**Fig 5:**
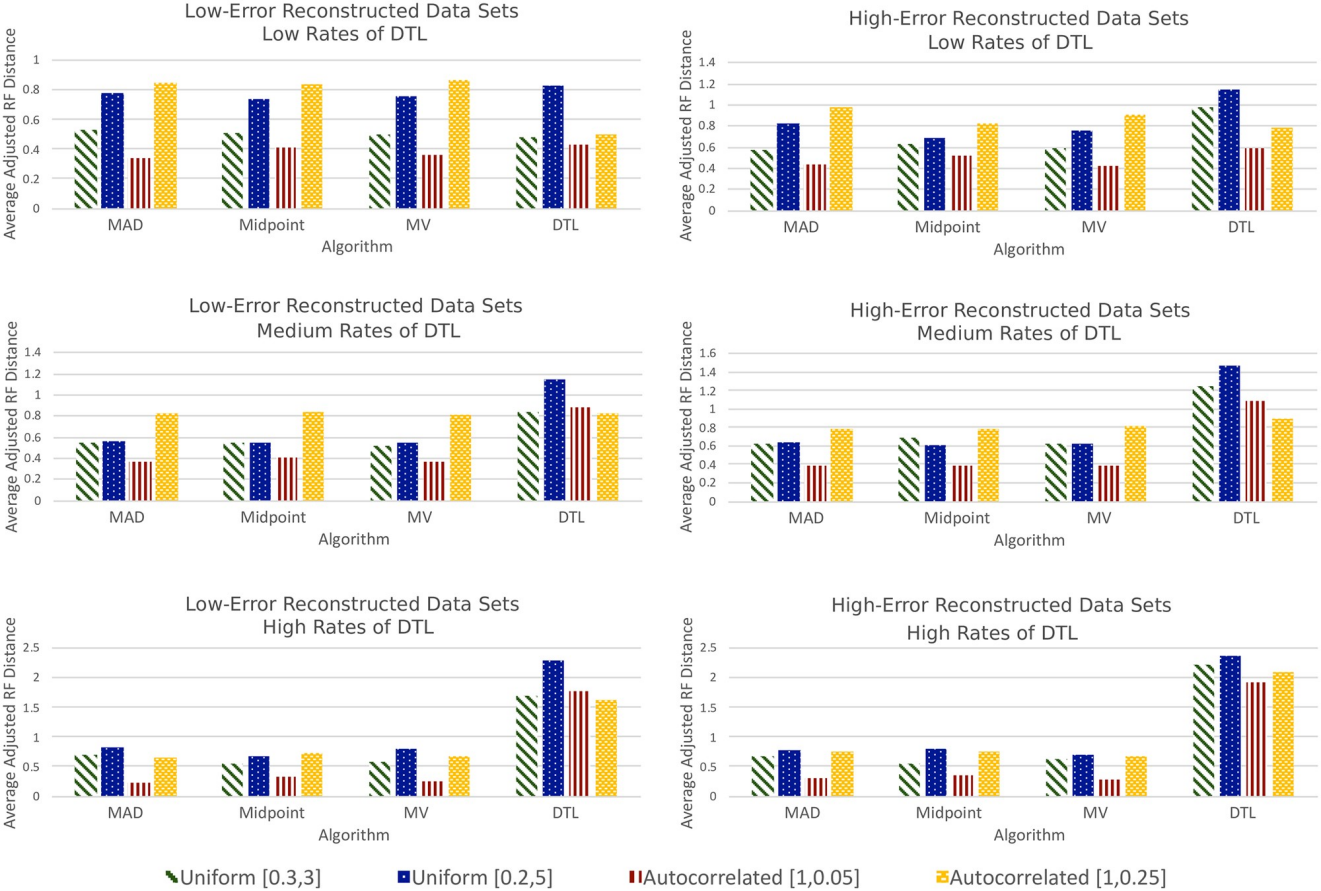
Results on reconstructed baseline data sets. The plots on the left show, for each rooting method and each of the 12 low-error reconstructed baseline data sets, the average adjusted RF distances for the reconstructed gene trees compared to their corresponding true gene trees. The plots on the right display analogous information for the 12 high-error reconstructed baseline data sets. Note that the RF distances displayed in these plots measure both tree reconstruction error and rooting error. Corresponding normalized RF distances are reported in Tables A3 and A4 in S1 Appendix.

Fig 5 shows rooting accuracy on the 24 reconstructed baseline data sets. Recall that we can measure rooting accuracy for a rooted reconstructed gene tree *T*′, corresponding to the true rooted gene tree *T*, by computing the *adjusted RF distance* between them (given by *RF*(*T*, *T*′) − *URF*(*T*, *T*′)). Fig 5 shows these average adjusted RF distances, and Table 2 reports the average *URF*(*T*, *T*′) values for all reconstructed baseline data sets. Despite the high reconstruction error-rates in these data sets, with an average unrooted RF distance of 7.33 across the 12 low-error data sets and 11.1 across the 12 high-error data sets (Table 2), we found that MAD rooting, MV rooting, and midpoint rooting achieved similar rooting accuracies as on the true gene trees. Specifically, for the low-error data sets, the average adjusted RF distances for MAD rooting, MV rooting, and midpoint rooting across all four branch scalings are 0.625, 0.623, and 0.625 for the low-DTL data sets, 0.573, 0.563, and 0.585 for the medium-DTL data sets, and 0.6, 0.57, and 0.573 for the high-DTL data sets, respectively. Likewise, for the high-error data sets, the average adjusted RF distances for MAD rooting, MV rooting, and midpoint rooting across all four branch scalings are 0.7, 0.668, and 0.665 for the low-DTL data sets, 0.608, 0.615, and 0.615 for the medium-DTL data sets, and 0.623, 0.573, and 0.605 for the high-DTL data sets, respectively. These values are in line with RF distances observed for these methods on the corresponding true gene trees (baseline data sets). In contrast, DTL rooting was clearly affected by gene tree reconstruction error. For the low-error data sets, the average adjusted RF distances for DTL rooting (with default event costs) across all four branch scalings are 0.56 for the low-DTL data sets, 0.923 for the medium-DTL data sets, and 1.84 for the high-DTL data sets. For the high-error data sets, the corresponding numbers were 0.875 for the low-DTL data sets, 1.175 for the medium-DTL data sets, and 2.155 for the high-DTL data sets. Thus, DTL rooting still remains the best performing method for low-DTL data sets for the low-error gene trees, but its rooting accuracy clearly decreases as gene tree error increases. Consistent with previous results on true gene trees, we also find that the rooting accuracy of DTL rooting decreases rapidly as the rate of evolutionary events increases.

In contrast to the results on (true) baseline data sets, we found that a very small fraction of the reconstructed gene trees were rooted “correctly” (i.e., with an adjusted RF distance of zero) by any of the evaluated rooting methods. Specifically, for the low-error data sets, the average number of correctly inferred rootings for MAD rooting, MV rooting, midpoint rooting, and DTL rooting across all 12 baseline reconstructed data sets were 1.92, 1.92, 1.83, and 1.42, respectively, while for the high-error data sets these averages were 1.33, 1.33, 1.33, and 1.25, respectively. None of the methods inferred correct roots for more than 5% of the trees in any of the 12 low-error reconstructed data sets, and more than 4% for any of the 12 high-error reconstructed data sets. Normalized RF distances for all methods on the reconstructed baseline data sets are reported in Tables A3 and A4 in S1 Appendix.

#### Impact of event costs on DTL rooting

**Fig 6:**
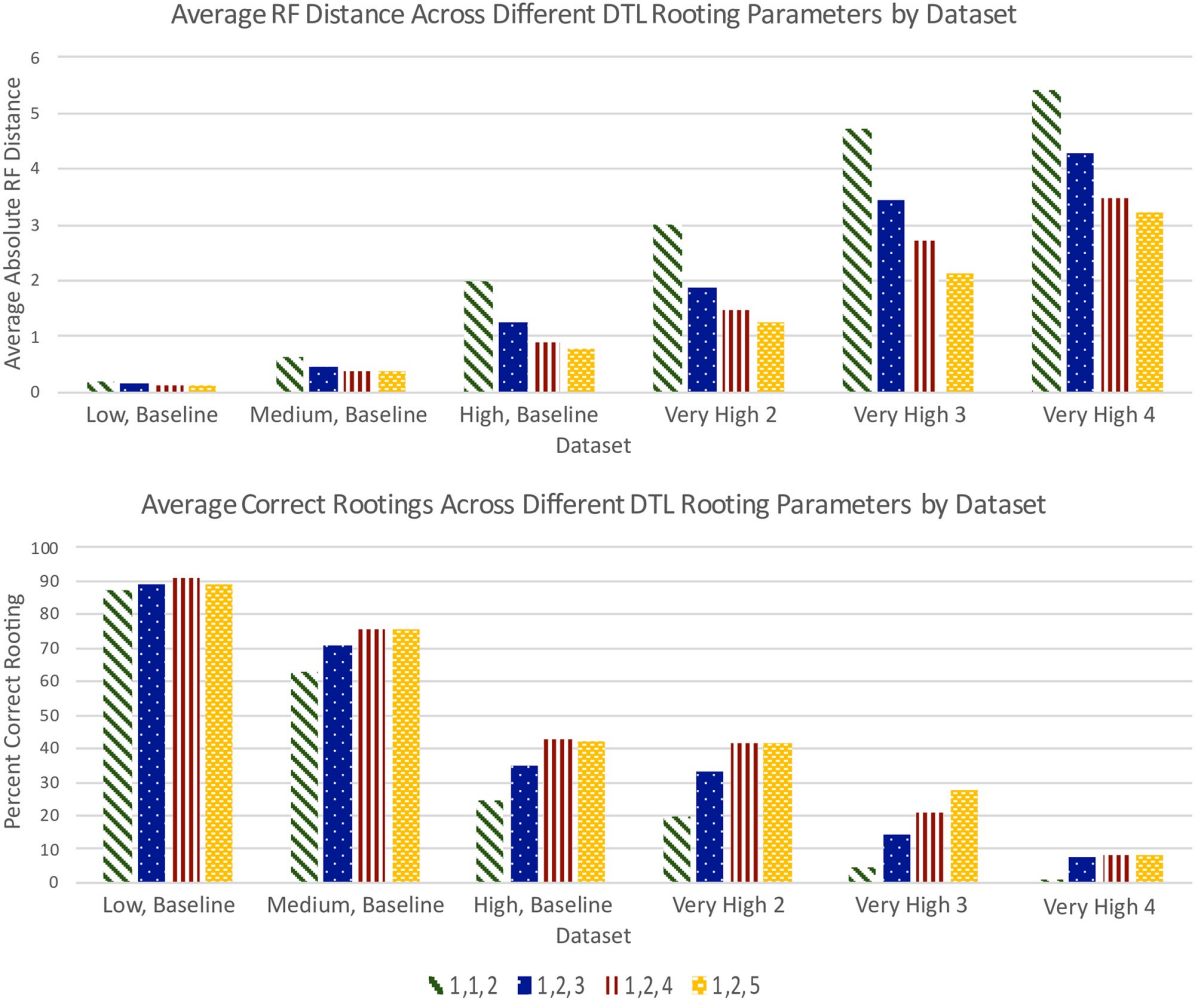
Impact of different event costs on DTL rooting accuracy. The plot on the top shows, for the different DTL rooting event costs used, the absolute average RF distances for the baseline and very high transfer rate data sets. The plot at the bottom shows the percentage of rootings inferred correctly on the same data sets.

Recall that all results reported above for DTL rooting use default event costs, i.e., 〈*loss, duplication, transfer*〉 costs 〈1, 2, 3〉. To assess the impact of varying these event costs on rooting accuracy, we applied DTL rooting to the baseline, very high transfer rate, and reconstructed baseline data sets using the following additional event costs: 〈1, 1, 2〉, 〈1, 2, 4〉, and 〈1, 2, 5〉. Fig 6 shows the rooting accuracies of DTL rooting with the different event costs on the 12 baseline data sets and the 12 very high transfer rate data sets (i.e., on true gene trees), and Fig A5 in S1 Appendix shows rooting accuracy results for the 24 reconstructed baseline data sets. As these figures show, there is a consistent and very clear trend across the true, low-error-, and high-error gene trees: Rooting accuracy improves substantially as transfer cost increases. For the baseline and very high transfer rate data sets (i.e., true gene trees), DTL rooting with event costs 〈1, 2, 5〉 even more strongly outperforms all other methods for the low- and medium-DTL data sets, but, despite significantly improved rooting accuracy, is still unable to outperform the other methods for high-DTL or any of the very high transfer rate data sets (Fig 6). The same trend is observed on the reconstructed baseline data sets as well, where DTL rooting with event costs 〈1, 2, 5〉 clearly outperforms all other methods on the low-DTL data sets for both the low-error and high-error gene trees, but performs worse for the medium- and high-DTL data sets (Fig A5 in S1 Appendix).

#### Interpretation of simulation study results

The results presented above show how the rooting accuracy of the considered methods is impacted by increasing amounts of deviation from the molecular clock, gene-level evolutionary events, and phylogenetic inference error. Note, however, that the amounts of molecular clock deviation and gene-level evolutionary events used in the simulation study cannot be directly compared to each other and, consequently, rooting accuracies of the reconciliation based methods cannot be directly compared with those of the branch-length based methods. Still, this simulation study reveals that (i) DTL rooting outperforms branch-length based methods for low to moderate rates of evolutionary events, even at relatively low (but not zero) levels of deviation from the molecular clock, and even when gene trees have some reconstruction error, (ii) DTL rooting becomes substantially more accurate when a higher cost is used for transfer events than is generally used by default, (iii) DTL rooting can lead to highly erroneous rootings when applied to gene trees with very high rates of horizontal gene transfer, (iv) ALE rooting performs consistently worse than DTL rooting, (v) MAD, MV, and midpoint rooting are generally unaffected by the rate of evolutionary events but are significantly affected by substitution rate variation across gene tree edges, (vi) MAD, MV, and midpoint rooting are generally robust to gene tree reconstruction error while DTL rooting is more sensitive to such errors, and (vii) MAD, MV, and midpoint rooting have similar accuracies on the simulated data sets.

### Results of empirical data analysis

Given the inherent uncertainty of phylogenetic reconstruction and lack of known rooting positions, it is not possible to directly assess the accuracy of the different rooting methods on the empirical data set. With this in mind, our empirical data set was carefully designed to be composed of taxa from two distinct and well characterized phyla: Alphaproteobacteria and Cyanobacteria. In line with the accepted evolutionary understanding of frequency of horizontal gene transfer being proportional to the evolutionary relatedness between taxa [43–45], we observe that regardless of the rooting method (DTL, MAD, MV, or midpoint rooting) used to root the gene trees, transfers between Alphaproteobacteria and Cyanobacteria accounted for less than 3% of inferred gene transfers among gene families present in both phyla. Since gene introgression between these phyla is so rare, the placement of the root in gene families conserved in both phyla likely coincides with taxonomic boundaries, i.e., is likely to be placed between Alphaproteobacteria and Cyanobacteria. Thus, on this data set, we assessed rooting similarities among methods by comparing the placement of each proposed gene tree root with the expected placement given the overall balance of the taxonomic distribution between Alphaproteobacteria and Cyanobacteria (0.2413), assuming the species tree root to be between them.

**Fig 7:**
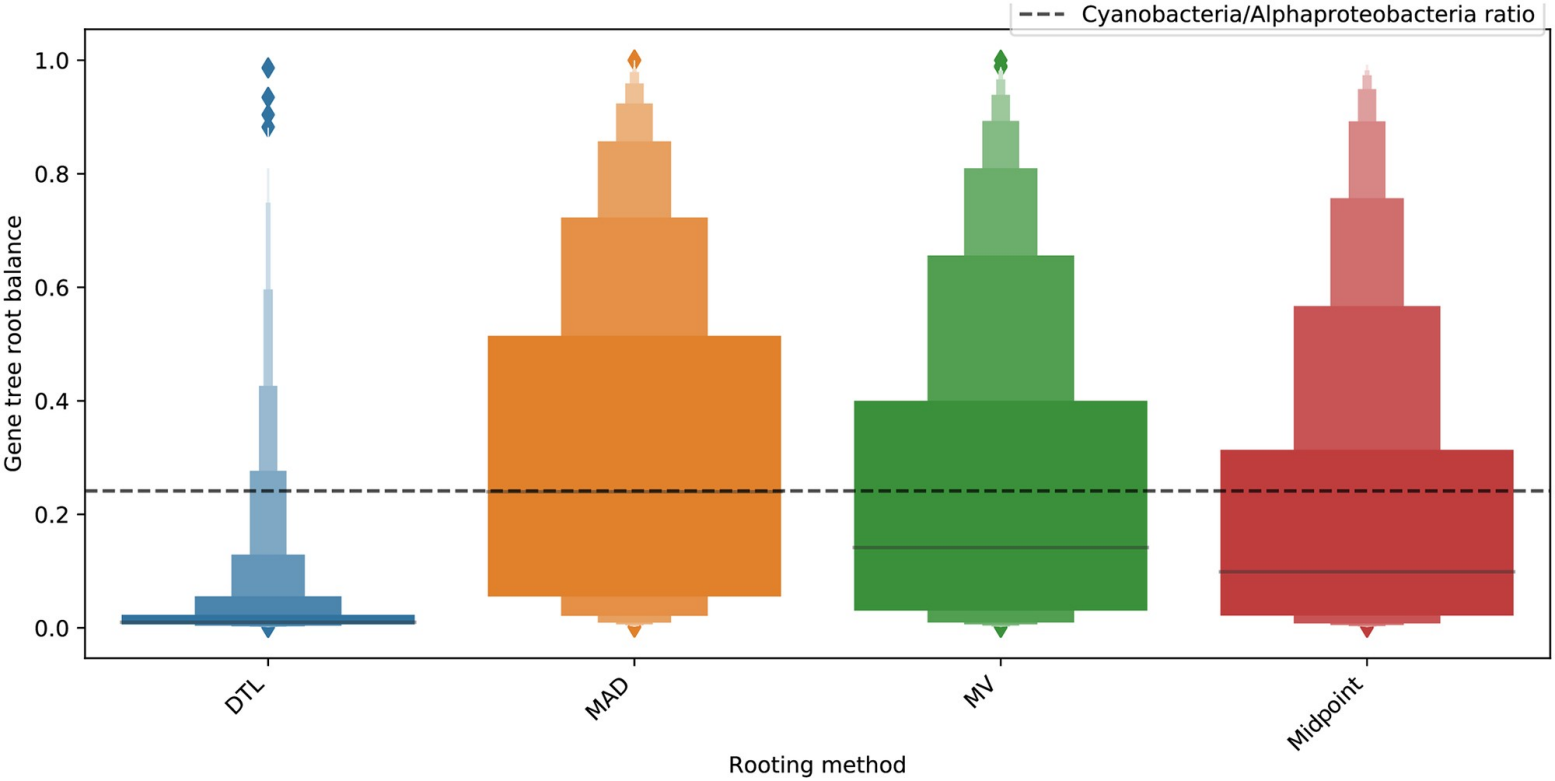
Letter-value plot of root balance distributions as inferred by DTL, MAD, MV, and midpoint rooting on all 3098 gene trees in our empirical data set. The dashed horizontal line represents the Cyanobacteria and Alphaproteobacteria ratio (0.2413). As the plot shows, MAD rooting yields root balances closest to the expected root balance. Results for all five rooting methods on the 3093 gene trees on which ALE rooting could be executed are shown in Fig A6 in S1 Appendix.

The empirical data set displays a substantial number of predicted duplication, transfer, and loss events, with the average ratio of number of events to number of tree tips being 0.433 for transfers and 0.196 for losses, across all 3098 assessed gene families. The number of duplications was negligible, with the ratio being 0.01. Out of the 3098 gene trees, 755 had a clean Cyanobacteria-Alphaproteobacteria split. The substantial number of transfer events is expected to have a negative impact on the accuracy of DTL rooting; indeed, very unbalanced root positions were frequently inferred, with an average root balance ratio of only 0.01 (see Fig 7). ALE rooting performed even worse, showing an average root balance ratio of 0.009 on the 3093 gene trees on which we could execute it (see Fig A6 in S1 Appendix). Among all five assessed root inference methods, MAD rooting generated root balances closer to the Cyanobacteria and Alphaproteobacteria ratio (0.2413) than any other method, with a mean balance of 0.2399 (Fig 7). MV rooting had an average root balance ratio of 0.1415 and mid-point rooting an average root balance of 0.0989. We also observed that root positions inferred using MAD rooting and MV rooting were very similar to each other, with both methods reporting identical root placements in 72.7% of cases. In contrast, MAD and midpoint rooting inferred identical roots only 36.2% of the time. MV and midpoint rooting inferred identical roots 40.3% of the time. ALE rooting and DTL rooting inferred identical roots 25.3% of the time and their rootings were almost entirely distinct from those inferred by the other three rooting methods. We point out that while ALE can simultaneously perform both error-correction and rooting of gene trees, in our analysis we used ALE only for gene tree rooting and did not invoke its error-correction functionality.

**Fig 8:**
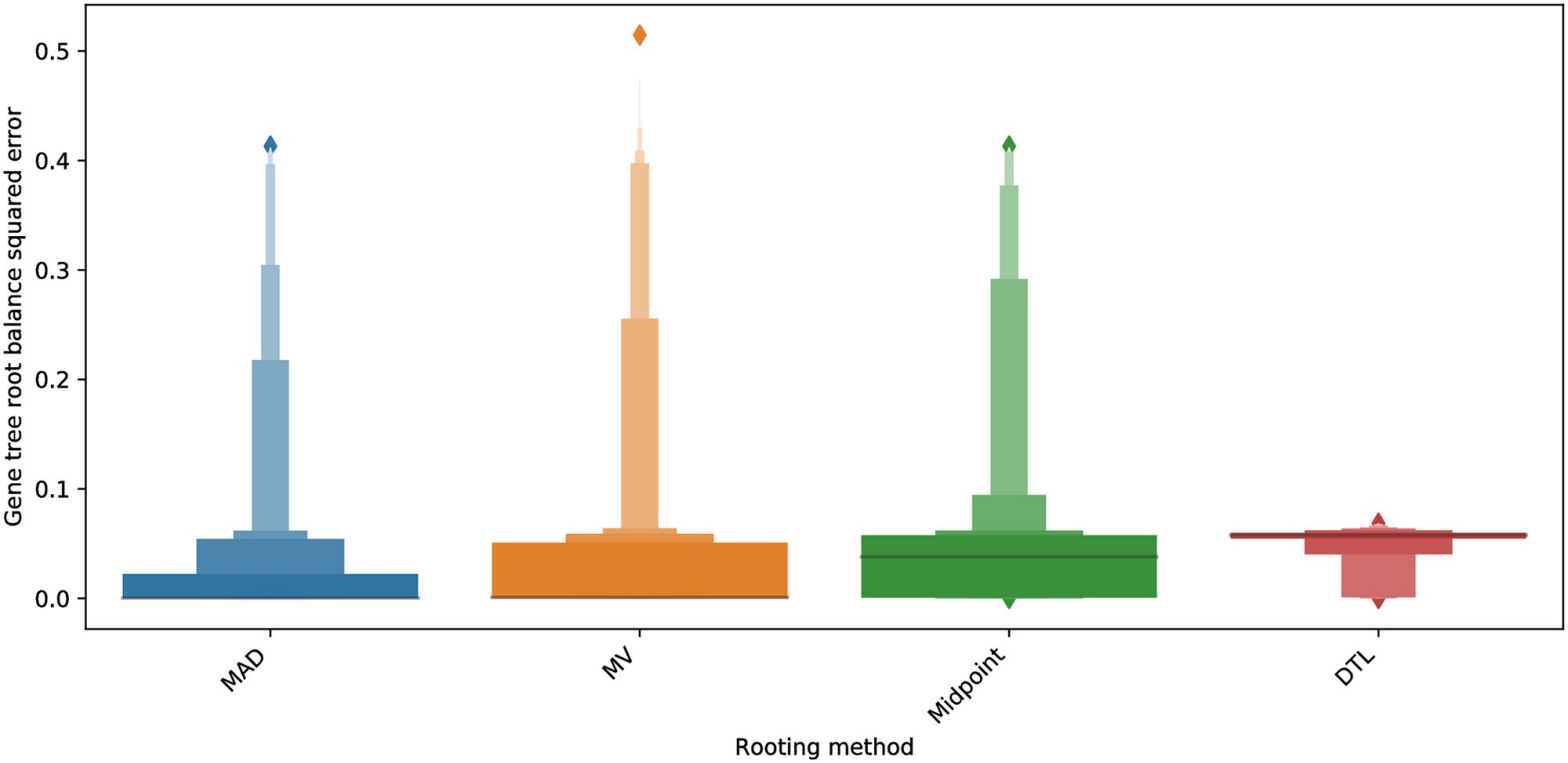
Letter-value plot of root balance squared error for the four rooting methods, DTL, MAD, MV, and midpoint rooting, on the 215 gene families in our empirical data set present in at least 90% of the Cyanobacteria and Alphaproteobacteria each. As the plot shows, MAD rooting yields the least mean squared error, with MV rooting a close second, while midpoint rooting and DTL rooting show significantly worse mean squared error.

Note that small gene trees with skewed species distributions can greatly impact the accuracy of our root balance results. We therefore repeated the above analysis with only the 215 gene families present in at least 90% of the Cyanobacteria and 90% of the Alphaproteobacteria and found that both MAD and MV showed average root balances of 0.2578 and 0.2321, which are almost identical to the Cyanobacteria and Alphaproteobacteria ratio (Fig A7 in S1 Appendix). The root balance ratio of midpoint rooting improved significantly to 0.1905 while DTL rootings remained highly skewed with an average root balance ratio of 0.0285 (Fig A7 in S1 Appendix). As before, ALE rooting performed slightly worse than DTL rooting on the 212 out of the 215 gene trees on which ALE could be executed (Fig A8 in S1 Appendix). We also measured the impact of using higher transfer costs for DTL rooting (event costs 1, 2, 5 for loss, duplication, and transfer, respectively) but found that this only slightly improved the root balance ratio (see “DTL custom” in Fig A7 in S1 Appendix). To distinguish between the relative accuracies of MAD and MV rooting, we further plotted the root balance ratio squared error for the different rooting methods for these 215 gene trees (Fig 8). MAD rooting showed the smallest mean squared error at 0.025, with MV rooting showing a larger mean squared error of 0.0338. Mean squared error for midpoint and DTL rooting was 0.0465 and 0.0519, respectively. As expected, on the subset of 212 gene trees, ALE rooting shows the worst mean squared error at 0.0521 (Fig A9 in S1 Appendix).

Overall, these results on empirical data supplement those from the simulation study and suggest that MAD rooting may be the most accurate rooting method, with an edge over MV rooting, on real biological gene trees with high or very high rates of evolutionary events.

#### Reconciliation costs of inferred rootings

Recall that DTL rooting finds a rooting with minimum duplication-transfer-loss (DTL) reconciliation cost. Thus, DTL rootings yield the lowest reconciliation costs, while MAD, MV, and midpoint rootings are expected to have higher reconciliation costs on our data set. Surprisingly, we noticed that root positions inferred by MAD rooting yielded average reconciliation costs only 2% greater than those corresponding to DTL rooting. We also compared the reconciliation costs of MAD, MV, and midpoint rootings against randomly chosen root positions and found that MAD rootings had lower reconciliation cost than 85% of the randomly rooted gene trees, while MV rooting and midpoint rooting had lower reconciliation cost than 84% and 83% of randomly rooted gene trees, respectively. These results indicate that correct rootings often have near-optimal reconciliation costs even for very high rates of evolutionary events.

#### Reconciliation-based rooting methods and gene tree error

Recall that results from the simulation study suggest that the rooting accuracy of reconciliation-based methods degrades rapidly with increasing phylogenetic inference error. The gene trees used in our empirical data analysis likely have high error rates, and this may partly explain why DTL rooting and ALE rooting performed poorly on the empirical data set. Several species-tree-aware gene tree reconstruction methods, such as ALE itself [27], TreeFix-DTL [34], and ecceTERA [29], have been developed over the past few years, and these methods have been shown to significantly reduce gene tree reconstruction error. The use of such species-tree-aware reconstruction methods may help to improve the accuracy of reconciliation-based, and perhaps even other, rooting methods.

## Discussion and conclusion

In this work, we have conducted a systematic study of the accuracy of five different gene tree rooting methods on prokaryotic gene trees. Our study is based on a large collection of both simulated and empirical gene trees and sheds light on the accuracy and relative merits of the different methods under a wide range of evolutionary conditions and reconstruction error. Results on the simulated and empirical data sets supplement each other and together provide several insights about the accuracies of the tested rooting methods. Specifically, our results suggest that: (1) Among the methods tested, DTL rooting and MAD rooting may be the two most effective methods for rooting prokaryotic gene families, with DTL rooting outperforming branch-length based methods for low to moderate rates of evolutionary events, even when there is relatively low (but not zero) molecular clock deviation, and with MAD rooting being most accurate for higher rates of evolutionary events, even at high levels of deviation from the molecular clock. (2) ALE rooting consistently performs worse than DTL rooting. (3) MV rooting and MAD rooting have similar rooting behaviors. (4) DTL rooting becomes substantially more accurate when a higher cost is used for transfer events than is generally used by default. (5) DTL rooting can be positively misleading, approaching the accuracy of random rooting, when applied to gene trees with very high rates of horizontal gene transfer. And (6), MAD, MV, and midpoint rooting are generally robust to gene tree reconstruction error and remain effective at rooting even when the gene trees under consideration have significant reconstruction error, while DTL rooting is more sensitive to such errors. In addition, our empirical analysis strongly suggests that MAD rooting outperforms MV and midpoint rooting but our simulation study finds these three methods to have comparable accuracy; this discrepancy suggests that using simulations alone may be insufficient and points to the need for careful empirical testing when assessing rooting methods.

This study suggests several promising directions for new and followup research. First, our results suggest that MAD, MV, and midpoint rooting are largely unaffected by frequencies of evolutionary events. This may be because under our simulation framework evolutionary events do not affect the substitution rate (or branch length) on the affected edges. It would be worth revisiting this aspect of the simulation study when more realistic models of gene family evolution that connect evolutionary events such as gene duplications and transfers with substitution rate changes are developed. Second, in this study we have used DTL rooting and ALE rooting with an undated species tree. This is because species trees can be extremely difficult to date accurately and so most species trees used for DTL or ALE rooting are expected to be undated in practice. However, using a dated species tree, when available, could help reduce instances of highly unbalanced rootings at very high transfer rates by preventing transfers that go backward in time. Third, we found that the accuracy of DTL rooting improves significantly as higher costs are assigned to transfer events. A more detailed theoretical or experimental study may provide insight into this phenomenon and help suggest the most accurate event costs to use under different evolutionary scenarios. Fourth, the results of our simulation and empirical studies are at odds regarding the relative accuracies of MAD, MV, and midpoint rooting, with simulations suggesting that all three methods have comparable accuracy but empirical analyses clearly favoring MAD rooting. It would be worthwhile to investigate underlying causes of this discrepancy. Fifth, we found that reconciliation-based rooting methods are particularly sensitive to gene tree error. Species-tree-aware gene tree reconstruction methods such as ALE [27], TreeFix-DTL [34], and ecceTERA [29], may help to improve the accuracy of reconciliation-based and other rooting methods by reconstructing more accurate gene trees. However, note that analyses performed on simulated true trees suggest that reconciliation-based methods encounter fundamental limitations even when gene trees are more accurate. Finally, our results suggest that a hybrid rooting method that searches for a rooting that is near-optimal in terms of DTL reconciliation cost, root balance, and MAD optimality may be able to outperform all existing rooting methods for prokaryotic gene trees.

## Supporting information

S1 Appendix Supplementary tables and figures, and SeqGen and RAxML commands. (PDF)

S1 Dataset Simulated data sets. (ZIP)

S2 Dataset Empirical data set. (ZIP)
