# Supplementary material for: Assessing the accuracy of phylogenetic rooting methods on prokaryotic gene families
Source: PLoS One. 2020 May 15;15(5):e0232950. doi: 10.1371/journal.pone.0232950 (PMC7228096; doi:10.1371/journal.pone.0232950)
Supplement: S1 Appendix — (PDF) [file pone.0232950.s001.pdf]

Appendix
Assessing the Accuracy of Phylogenetic Rooting Methods on Prokaryotic Gene Families

– Taylor Wade, L. Thiberio Rangel, Soumya Kundu, Gregory P. Fournier and Mukul S. Bansal

Table A1. Rooting accuracy on baseline simulated data sets. Average absolute and normalized Robinson-Foulds distances for the five rooting methods on the 12 sets of baseline gene trees. Results are averaged across the 100 gene trees in each set.

Data set	MAD Abs. RFD	MAD Norm. RFD	MV Abs. RFD	MV Norm. RFD	MP Abs. RFD	MP Norm RFD
Low-DTL, Uniform [0.3, 3]	0.53	0.00455	0.49	0.00431	0.48	0.00402
Medium-DTL, Uniform [0.3, 3]	0.6	0.00878	0.59	0.00866	0.61	0.00905
High-DTL, Uniform [0.3, 3]	0.61	0.00803	0.54	0.00754	0.54	0.00762
Low-DTL, Uniform [0.2, 5]	0.76	0.00665	0.72	0.00628	0.71	0.0063
Medium-DTL, Uniform [0.2, 5]	0.62	0.00891	0.62	0.00908	0.59	0.00819
High-DTL, Uniform [0.2, 5]	0.85	0.00933	0.81	0.00867	0.76	0.00794
Low-DTL, Auto. [1, 0.05]	0.28	0.00248	0.27	0.00239	0.31	0.00274
Medium-DTL, Auto. [1, 0.05]	0.37	0.00617	0.37	0.00617	0.33	0.00581
High-DTL, Auto. [1, 0.05]	0.26	0.00199	0.25	0.00191	0.24	0.00181
Low-DTL, Auto. [1, 0.25]	0.89	0.00736	0.84	0.00693	0.85	0.00712
Medium-DTL, Auto. [1, 0.25]	0.82	0.00796	0.79	0.00768	0.81	0.00767
High-DTL, Auto. [1, 0.25]	0.74	0.00668	0.69	0.00631	0.71	0.0066

Data set	DTL Abs. RFD	DTL Norm. RFD	ALE Abs. RFD	ALE Norm RFD
Low-DTL, Uniform [0.3, 3]	0.16	0.00129	0.23	0.00191
Medium-DTL, Uniform [0.3, 3]	0.48	0.00401	0.84	0.00722
High-DTL, Uniform [0.3, 3]	1.21	0.0162	1.43	0.01957
Low-DTL, Uniform [0.2, 5]	0.18	0.00161	0.2	0.00161
Medium-DTL, Uniform [0.2, 5]	0.46	0.00452	0.79	0.0101
High-DTL, Uniform [0.2, 5]	1.28	0.01869	1.69	0.0224
Low-DTL, Auto. [1, 0.05]	0.15	0.00131	0.24	0.00194
Medium-DTL, Auto. [1, 0.05]	0.44	0.00373	0.063	0.0056
High-DTL, Auto. [1, 0.05]	1.24	0.01692	1.49	0.02028
Low-DTL, Auto. [1, 0.25]	0.18	0.00178	0.25	0.00205
Medium-DTL, Auto. [1, 0.25]	0.46	0.00384	0.78	0.00636
High-DTL, Auto. [1, 0.25]	1.26	0.0179	1.33	0.01756

Seq-Gen and RAxML commands used

We used the following commands to generate aligned nucleotide sequences for each gene tree from the baseline data set.
For the 1000nt sequences:

```
seq-gen -mGTR -l1000 -n1 -s1 -a1 -g4 -or -q < inputTree.newick > output.fasta
```

For the 500nt sequences:

```
seq-gen -mGTR -l500 -n1 -s1 -a1 -g4 -or -q < inputTree.newick > output.fasta
```

We used the following command to generate the reconstructed gene tree for each set of aligned sequences generated using Seq-Gen.

```
raxmlHPC-SSE3 -f d -m GTRCAT -p 12345 -s file.fasta
```

Table A2. Distribution of rooting accuracies on baseline simulated data sets. The three quartiles of absolute Robinson-Foulds distances for the five rooting methods on the 12 sets of baseline gene trees.

Data set	MAD Q_1	MAD Q_2	MAD Q_3	MV Q_1	MV Q_2	MV Q_3
Low-DTL, Uniform $[0.3, 3]$	0	0	1	0	0	1
Medium-DTL, Uniform $[0.3, 3]$	0	0	1	0	0	1
High-DTL, Uniform $[0.3, 3]$	0	0	1	0	0	1
Low-DTL, Uniform $[0.2, 5]$	0	1	1	0	1	1
Medium-DTL, Uniform $[0.2, 5]$	0	0	1	0	0	1
High-DTL, Uniform $[0.2, 5]$	0	0	1	0	0	1
Low-DTL, Auto. $[1, 0.05]$	0	0	1	0	0	0.25
Medium-DTL, Auto. $[1, 0.05]$	0	0	1	0	0	1
High-DTL, Auto. $[1, 0.05]$	0	0	0	0	0	0
Low-DTL, Auto. $[1, 0.25]$	0	0	1	0	0	1
Medium-DTL, Auto. $[1, 0.25]$	0	0	1	0	0	1
High-DTL, Auto. $[1, 0.25]$	0	0	1	0	0	1

Data set	MP Q_1	MP Q_2	MP Q_3	DTL Q_1	DTL Q_2	DTL Q_3
Low-DTL, Uniform $[0.3, 3]$	0	0	1	0	0	0
Medium-DTL, Uniform $[0.3, 3]$	0	0	1	0	0	1
High-DTL, Uniform $[0.3, 3]$	0	0	1	0	1	2
Low-DTL, Uniform $[0.2, 5]$	0	1	1	0	0	0
Medium-DTL, Uniform $[0.2, 5]$	0	0	1	0	0	1
High-DTL, Uniform $[0.2, 5]$	0	0	1	0	1	2
Low-DTL, Auto. $[1, 0.05]$	0	0	1	0	0	0
Medium-DTL, Auto. $[1, 0.05]$	0	0	1	0	0	1
High-DTL, Auto. $[1, 0.05]$	0	0	0	0	1	2
Low-DTL, Auto. $[1, 0.25]$	0	1	1	0	0	0
Medium-DTL, Auto. $[1, 0.25]$	0	0	1	0	0	1
High-DTL, Auto. $[1, 0.25]$	0	0	1	0	1	2

Data set	ALE Q_1	ALE Q_2	ALE Q_3
Low-DTL, Uniform $[0.3, 3]$	0	0	0
Medium-DTL, Uniform $[0.3, 3]$	0	0	1
High-DTL, Uniform $[0.3, 3]$	0	1	2
Low-DTL, Uniform $[0.2, 5]$	0	0	0
Medium-DTL, Uniform $[0.2, 5]$	0	0	1
High-DTL, Uniform $[0.2, 5]$	0	1	2
Low-DTL, Auto. $[1, 0.05]$	0	0	0
Medium-DTL, Auto. $[1, 0.05]$	0	0	1
High-DTL, Auto. $[1, 0.05]$	0	1	2
Low-DTL, Auto. $[1, 0.25]$	0	0	0
Medium-DTL, Auto. $[1, 0.25]$	0	0	1
High-DTL, Auto. $[1, 0.25]$	0	1	2

Table A3. Rooting accuracy on low-error reconstructed baseline data sets. Average adjusted and normalized-adjusted Robinson-Foulds distances for the five rooting methods on the 12 sets of low-error baseline reconstructed gene trees. Results are averaged across the 100 gene trees in each set.

Data set	MAD Adj. RFD	MAD Norm-Adj RFD	MV Adj. RFD	MV Norm-Adj RFD	MP Adj. RFD	MP Norm-Adj RFD
Low-DTL, Uniform $[0.3, 3]$	0.53	0.00449	0.5	0.00447	0.51	0.00448
Medium-DTL, Uniform $[0.3, 3]$	0.55	0.00812	0.52	0.00787	0.55	0.00798
High-DTL, Uniform $[0.3, 3]$	0.69	0.00817	0.58	0.0074	0.56	0.0075
Low-DTL, Uniform $[0.2, 5]$	0.78	0.00669	0.76	0.00657	0.74	0.00647
Medium-DTL, Uniform $[0.2, 5]$	0.56	0.00833	0.55	0.00817	0.55	0.00759
High-DTL, Uniform $[0.2, 5]$	0.82	0.00942	0.79	0.0088	0.68	0.00722
Low-DTL, Auto. $[1, 0.05]$	0.34	0.00296	0.36	0.00315	0.41	0.00362
Medium-DTL, Auto. $[1, 0.05]$	0.36	0.00652	0.37	0.00655	0.4	0.0067
High-DTL, Auto. $[1, 0.05]$	0.23	0.00189	0.24	0.00194	0.32	0.00263
Low-DTL, Auto. $[1, 0.25]$	0.85	0.00713	0.87	0.00733	0.84	0.0070
Medium-DTL, Auto. $[1, 0.25]$	0.82	0.00845	0.81	0.00815	0.84	0.00843
High-DTL, Auto. $[1, 0.25]$	0.66	0.00618	0.67	0.00627	0.73	0.00702

Data set	DTL Adj. RFD	DTL Norm-Adj RFD
Low-DTL, Uniform $[0.3, 3]$	0.48	0.00437
Medium-DTL, Uniform $[0.3, 3]$	0.84	0.00883
High-DTL, Uniform $[0.3, 3]$	1.69	0.0225
Low-DTL, Uniform $[0.2, 5]$	0.83	0.0075
Medium-DTL, Uniform $[0.2, 5]$	1.15	0.01242
High-DTL, Uniform $[0.2, 5]$	2.29	0.02565
Low-DTL, Auto. $[1, 0.05]$	0.43	0.00393
Medium-DTL, Auto. $[1, 0.05]$	0.88	0.00934
High-DTL, Auto. $[1, 0.05]$	1.76	0.02339
Low-DTL, Auto. $[1, 0.25]$	0.5	0.00427
Medium-DTL, Auto. $[1, 0.25]$	0.82	0.00793
High-DTL, Auto. $[1, 0.25]$	1.61	0.02004

Table A4. Rooting accuracy on high-error reconstructed baseline data sets. Average adjusted and normalized-adjusted Robinson-Foulds distances for the five rooting methods on the 12 sets of high-error baseline reconstructed gene trees. Results are averaged across the 100 gene trees in each set.

Data set	MAD Adj. RFD	MAD Norm-Adj RFD	MV Adj. RFD	MV Norm-Adj RFD	MP Adj. RFD	MP Norm-Adj RFD
Low-DTL, Uniform $[0.3, 3]$	0.57	0.0049	0.58	0.00513	0.63	0.00549
Medium-DTL, Uniform $[0.3, 3]$	0.62	0.00927	0.63	0.00931	0.68	0.00998
High-DTL, Uniform $[0.3, 3]$	0.68	0.00827	0.63	0.00754	0.54	0.0065
Low-DTL, Uniform $[0.2, 5]$	0.82	0.0071	0.76	0.0066	0.68	0.00589
Medium-DTL, Uniform $[0.2, 5]$	0.64	0.0095	0.63	0.00941	0.6	0.00857
High-DTL, Uniform $[0.2, 5]$	0.78	0.0085	0.71	0.0073	0.8	0.0074
Low-DTL, Auto. $[1, 0.05]$	0.43	0.00395	0.42	0.00389	0.52	0.00467
Medium-DTL, Auto. $[1, 0.05]$	0.38	0.00619	0.39	0.00629	0.39	0.00609
High-DTL, Auto. $[1, 0.05]$	0.29	0.00243	0.27	0.00216	0.34	0.00277
Low-DTL, Auto. $[1, 0.25]$	0.98	0.00831	0.91	0.00771	0.83	0.00716
Medium-DTL, Auto. $[1, 0.25]$	0.79	0.00766	0.81	0.00791	0.79	0.00743
High-DTL, Auto. $[1, 0.25]$	0.74	0.00647	0.68	0.00599	0.74	0.00676

Data set	DTL Adj. RFD	DTL Norm-Adj RFD
Low-DTL, Uniform $[0.3, 3]$	0.98	0.00863
Medium-DTL, Uniform $[0.3, 3]$	1.24	0.01295
High-DTL, Uniform $[0.3, 3]$	2.22	0.02755
Low-DTL, Uniform $[0.2, 5]$	1.15	0.01006
Medium-DTL, Uniform $[0.2, 5]$	1.47	0.01594
High-DTL, Uniform $[0.2, 5]$	2.38	0.02643
Low-DTL, Auto. $[1, 0.05]$	0.59	0.00517
Medium-DTL, Auto. $[1, 0.05]$	1.09	0.01162
High-DTL, Auto. $[1, 0.05]$	1.92	0.02214
Low-DTL, Auto. $[1, 0.25]$	0.78	0.00684
Medium-DTL, Auto. $[1, 0.25]$	0.9	0.00877
High-DTL, Auto. $[1, 0.25]$	2.1	0.02221

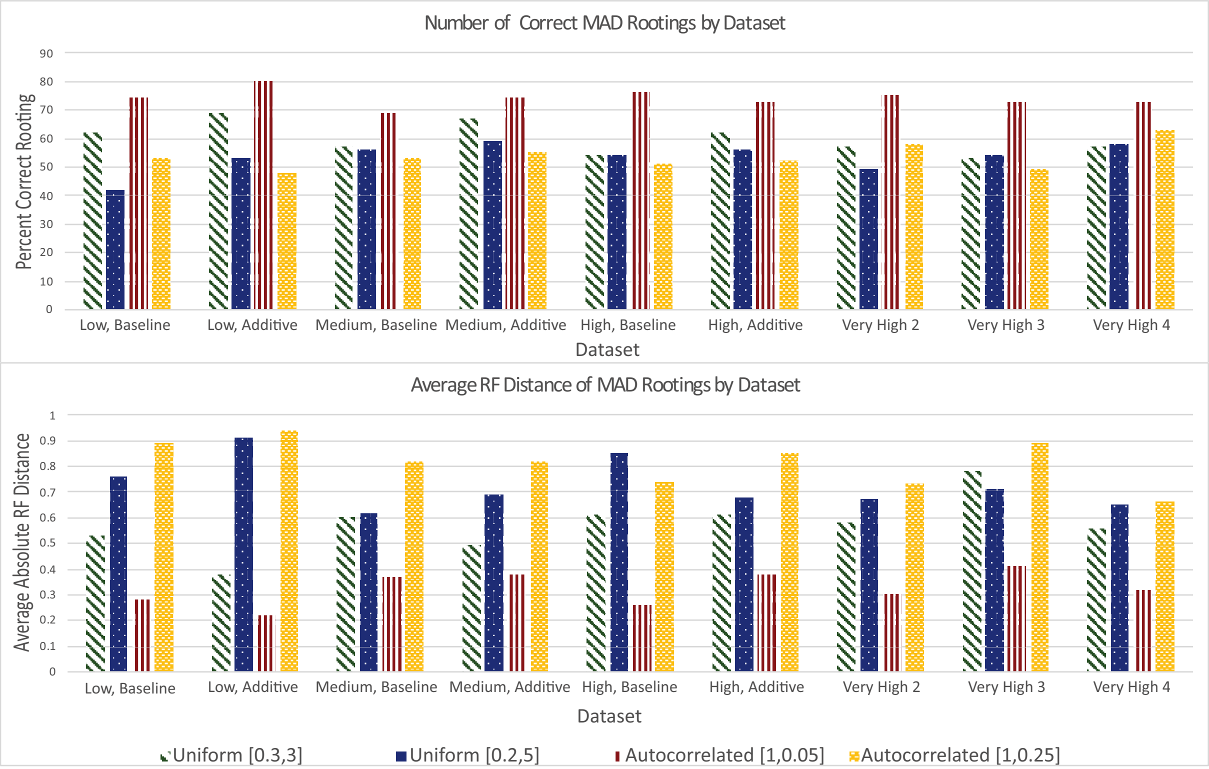

Fig. A1. Performance of MAD rooting across each of the 36 sets of true simulated gene trees. The top half shows, for each set of 100 gene trees, the percent of gene trees for which the root was inferred correctly. The bottom half shows, for each set of gene trees, the absolute average RF distance of the inferred rooted gene trees to the correctly rooted gene trees. As the plots show, the root inference accuracy of MAD rooting was unaffected by increasing rates of evolutionary events or by the relative abundance of additive and replacing transfers. However, rooting accuracy degrades rapidly as mutation rate variation (or scaling) across branches increases, with Autocorrelated [1,0.25] scaling resulting in the worst accuracy.

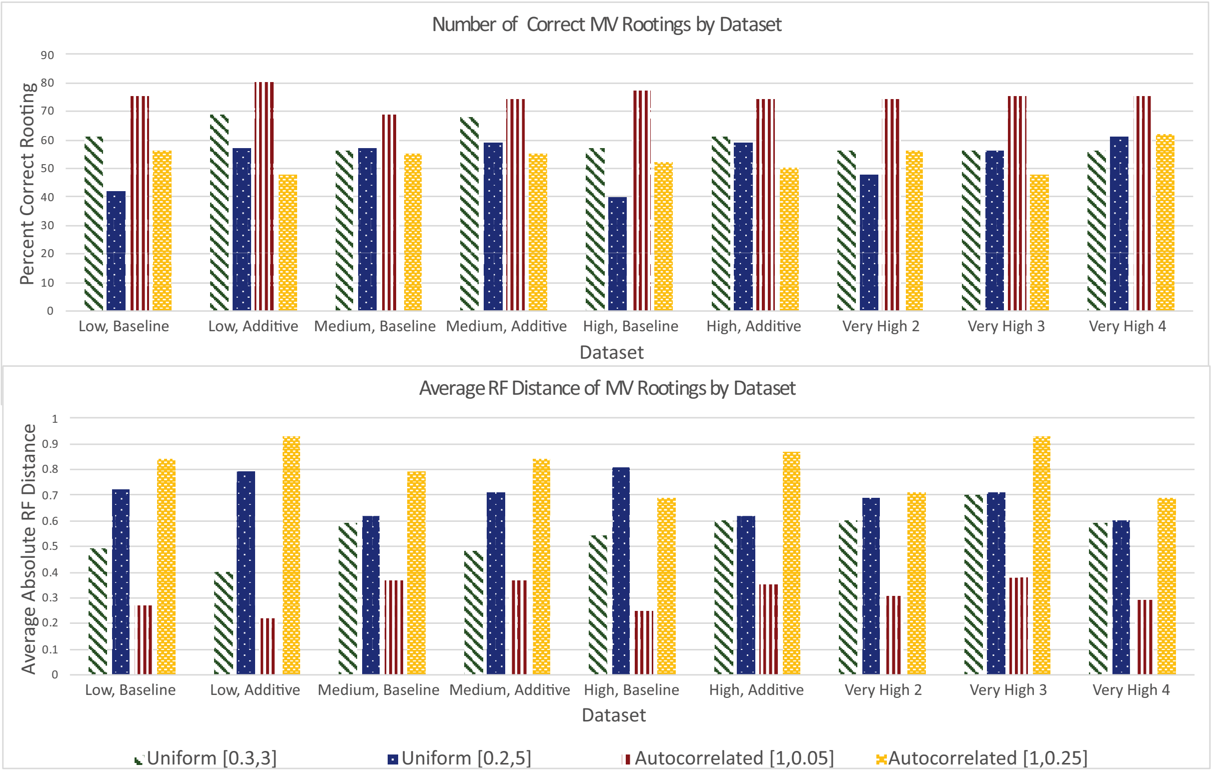

Fig. A2. Performance of MV rooting across each of the 36 sets of true simulated gene trees. The top half shows, for each set of 100 gene trees, the percent of gene trees for which the root was inferred correctly. The bottom half shows, for each set of gene trees, the absolute average RF distance of the inferred rooted gene trees to the correctly rooted gene trees. As the plots show, the root inference accuracy of MV rooting was unaffected by increasing rates of evolutionary events or by the relative abundance of additive and replacing transfers. However, rooting accuracy degrades rapidly as mutation rate variation (or scaling) across branches increases, with Autocorrelated [1,0.25] scaling resulting in the worst accuracy.

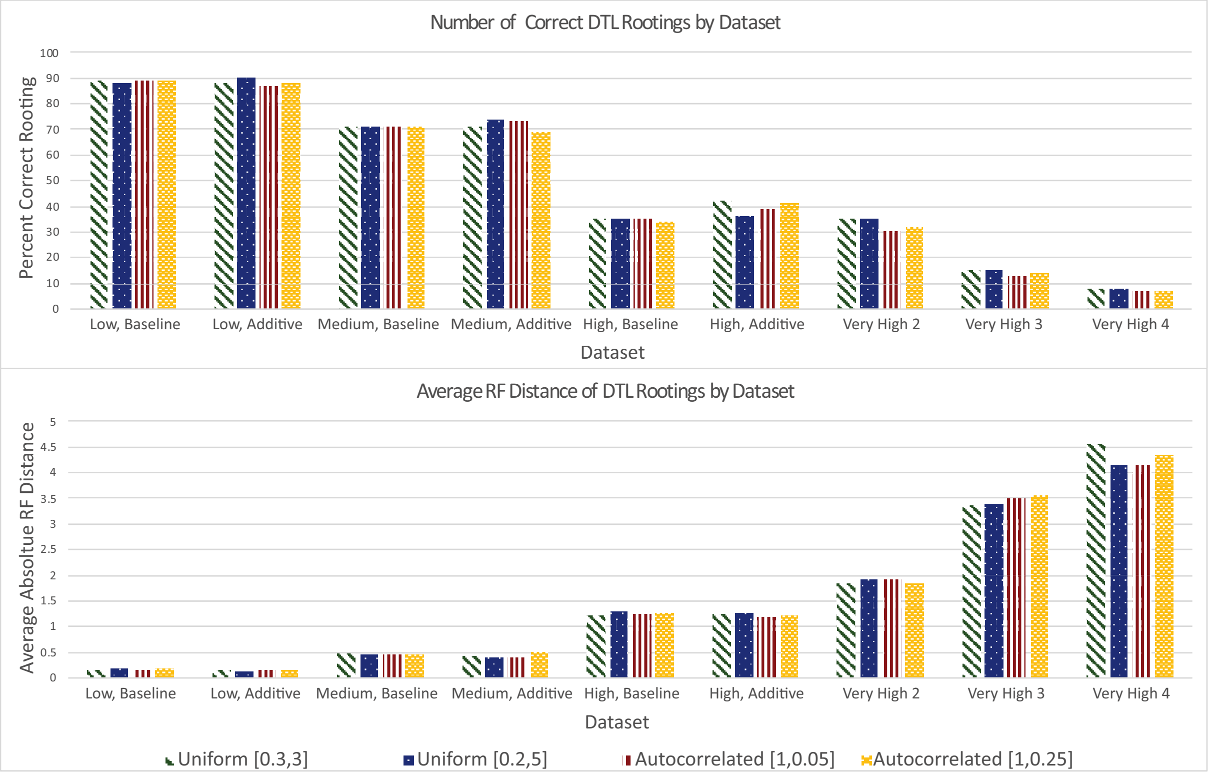

Fig. A3. Performance of DTL rooting across each of the 36 sets of true simulated gene trees. The top half shows, for each set of 100 gene trees, the percent of gene trees for which the root was inferred correctly. The bottom half shows, for each set of gene trees, the absolute average RF distance of the inferred rooted gene trees to the correctly rooted gene trees. As expected, the root inference accuracy of DTL rooting is unaffected by mutation rate variation (or scaling) across branches or by the relative abundance of additive and replacing transfers. However, the accuracy of DTL rooting degrades rapidly as the rate of evolutionary events (especially transfers) increases.

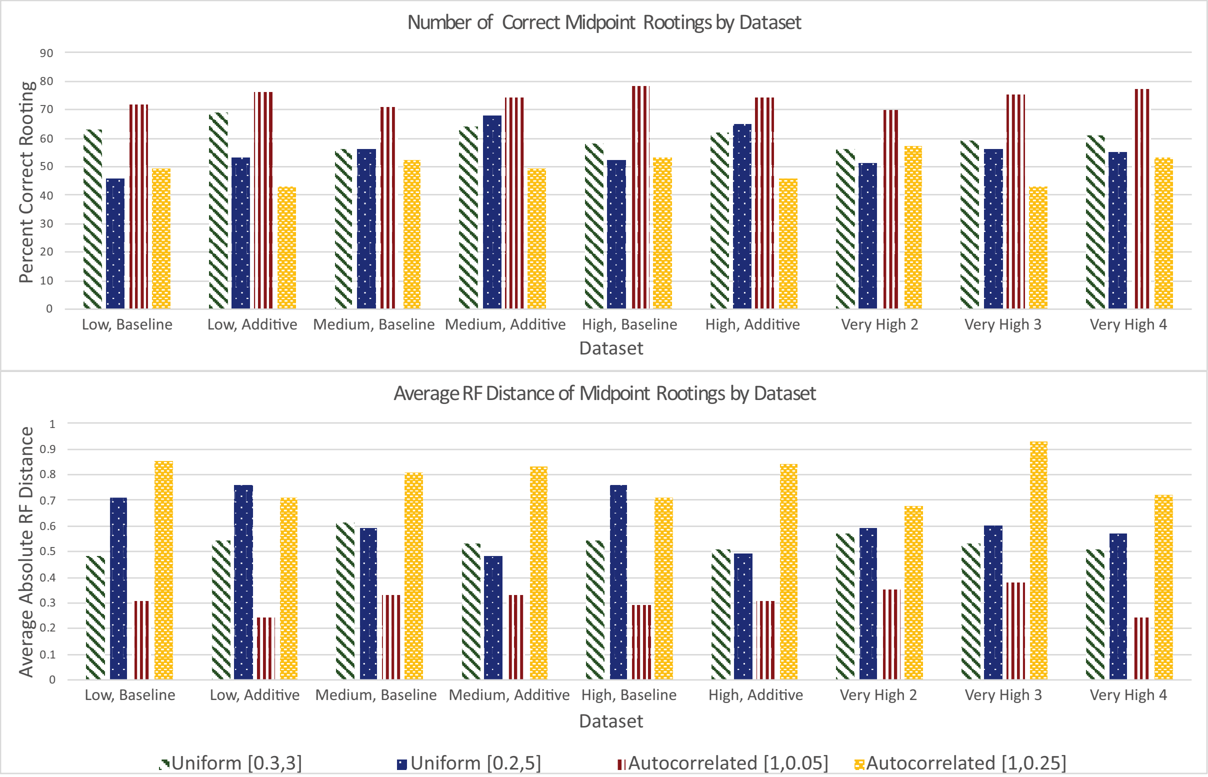

Fig. A4. Performance of midpoint rooting across each of the 36 sets of true simulated gene trees. The top half shows, for each set of 100 gene trees, the percent of gene trees for which the root was inferred correctly. The bottom half shows, for each set of gene trees, the absolute average RF distance of the inferred rooted gene trees to the correctly rooted gene trees. As the plots show, the root inference accuracy of midpoint rooting was unaffected by increasing rates of evolutionary events or by the relative abundance of additive and replacing transfers. Rooting accuracy degrades rapidly as mutation rate variation (or scaling) across branches increases, with Autocorrelated [1,0.25] scaling resulting in the worst accuracy.

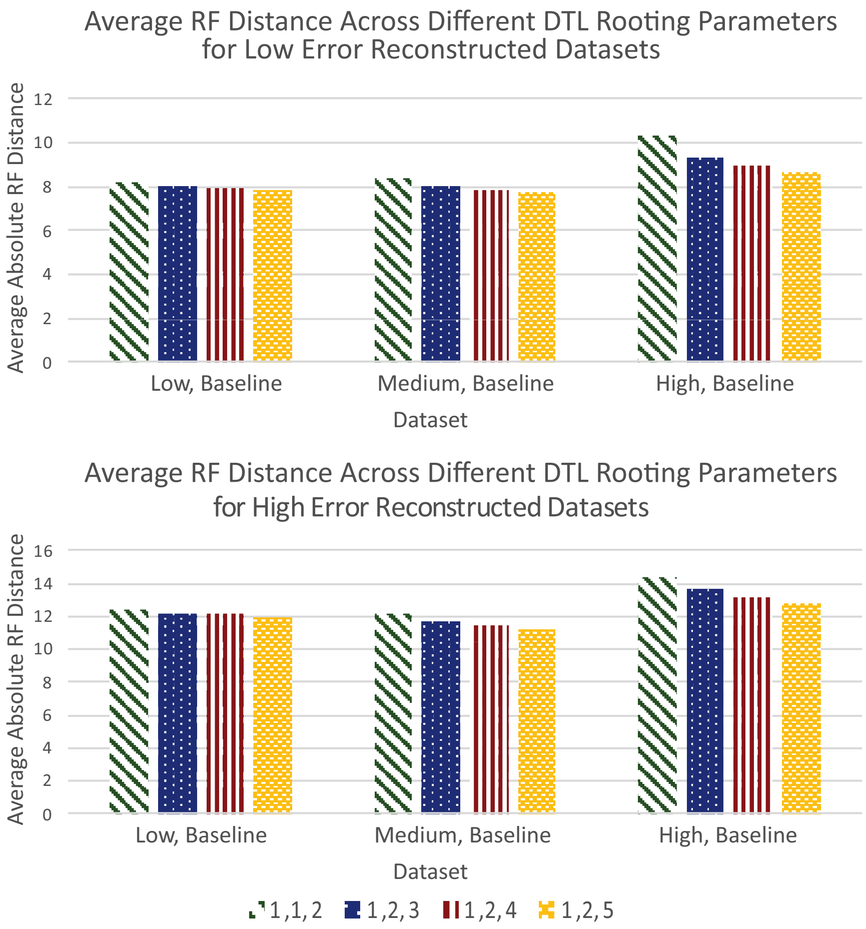

Fig. A5. Impact of different event costs on DTL rooting accuracy on reconstructed data sets. The plot on the top shows, for the different DTL rooting event costs used, the absolute average RF distances for the low-error reconstructed gene trees. The plot at the bottom displays analogous information for the high-error reconstructed gene trees.

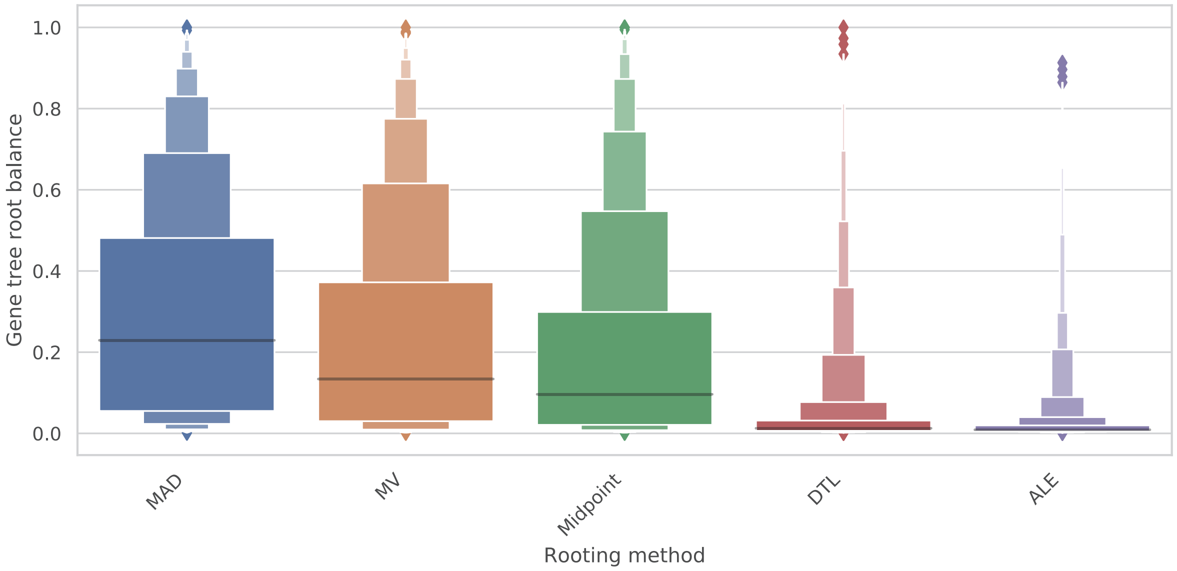

Fig. A6. Letter-value plot of root balance distributions as inferred by the five rooting methods, DTL, MAD, MV, midpoint, and ALE rooting, on the 3093 gene families in our empirical data set on which ALE rooting could be executed. As the plot shows, MAD rooting yields root balances closest to the expected root balance, while ALE rooting yields the most skewed root balances.

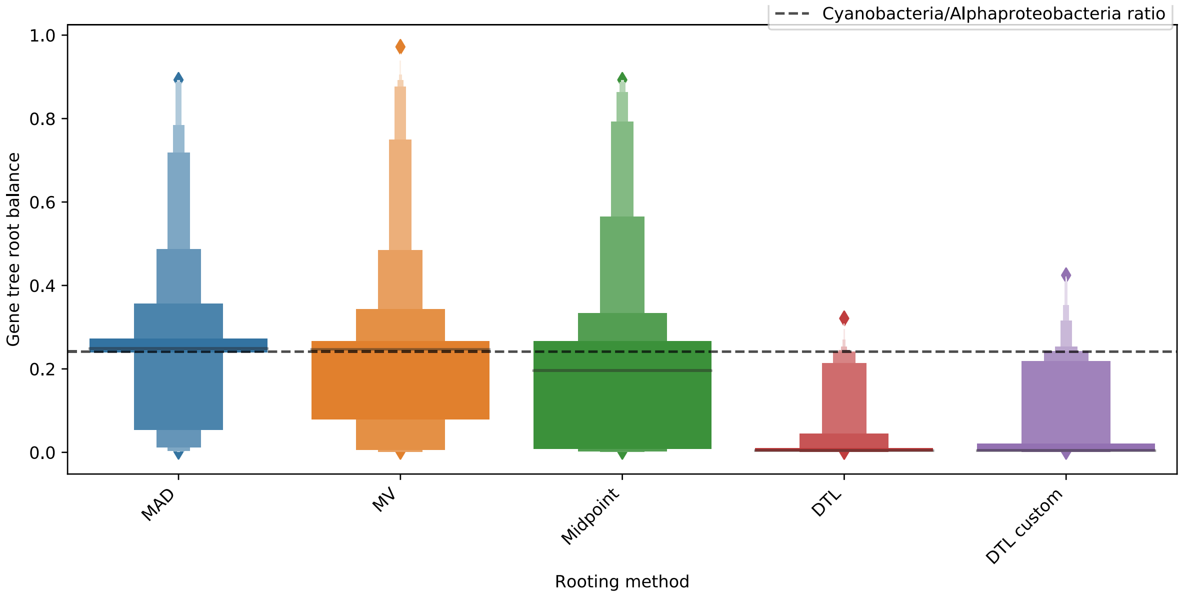

Fig. A7. Letter-value plot of root balance distributions as inferred by the four rooting methods, DTL, MAD, MV, and midpoint rooting, on the 215 gene families in our empirical data set present in at least 90% of the Cyanobacteria and Alphaproteobacteria each. The dashed horizontal line represents the Cyanobacteria and Alphaproteobacteria ratio (0.2413). As the plot shows, MAD and MV rooting yield root balances closest to the expected root balance, while DTL rooting yields the most skewed root balances. In this plot, "DTL" corresponds to DTL rooting executed with default event costs of 1 for loss, 2 for duplication, and 3 for horizontal transfer, while "DTL custom" corresponds to event costs of 1 for loss, 2 for duplication, and 5 for horizontal transfer. Results for all five rooting methods on the 212 out of these 215 gene trees on which ALE rooting could be executed are shown in Figure A8.

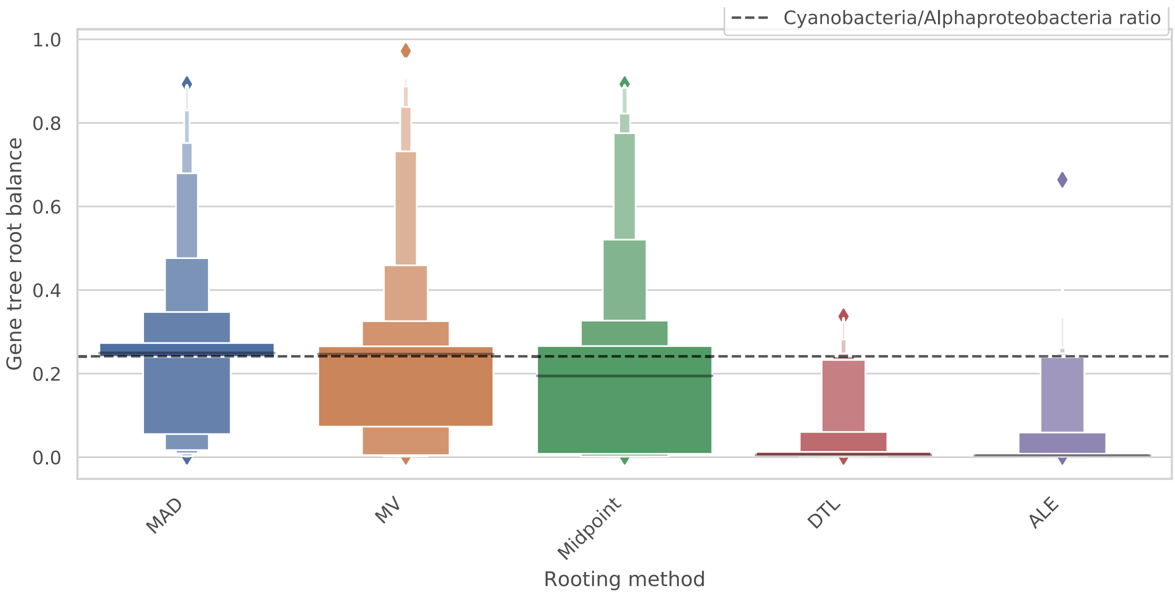

Fig. A8. Letter-value plot of root balance distributions as inferred by five rooting methods, DTL, MAD, MV, midpoint, and ALE rooting, on the 212 gene families in our empirical data set present in at least 90% of the Cyanobacteria and Alphaproteobacteria each on which ALE rooting could be executed. The dashed horizontal line represents the Cyanobacteria and Alphaproteobacteria ratio (0.2413). As the plot shows, MAD and MV rooting yield root balances closest to the expected root balance, while ALE rooting yields the most skewed root balances.

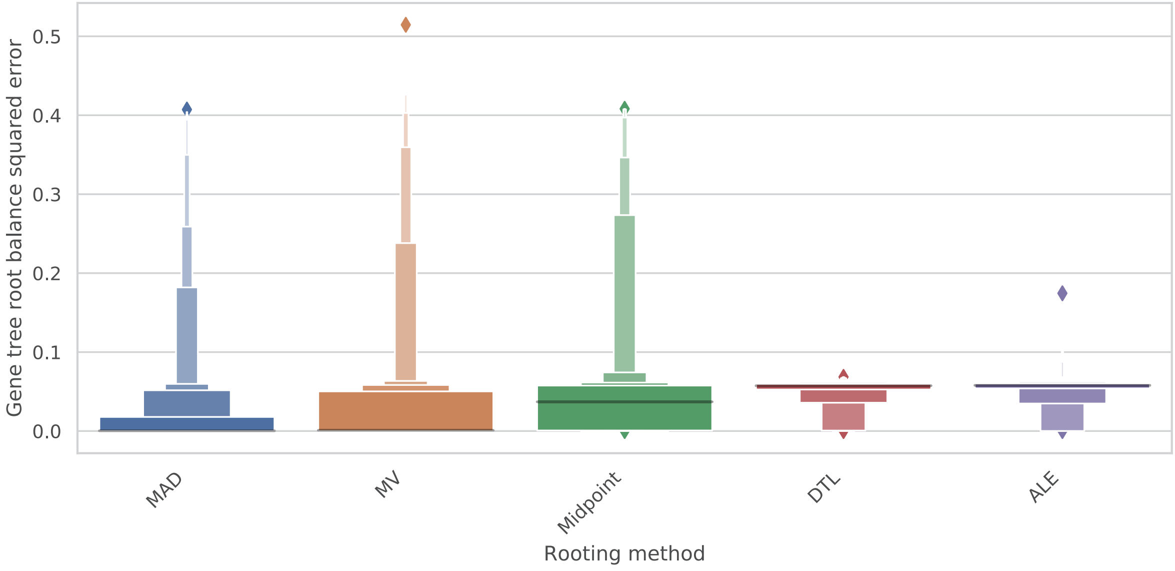

Fig. A9. Letter-value plot of root balance squared error for the five rooting methods, DTL, MAD, MV, midpoint, and ALE rooting, on the 212 gene families in our empirical data set present in at least 90% of the Cyanobacteria and Alphaproteobacteria each on which ALE rooting could be executed. As the plot shows, MAD rooting yields the least mean squared error, with MV rooting a close second, while ALE rooting and DTL rooting show significantly worse mean squared errors.
